# Reduced Expression of *CbUFO* Is Associated with the Phenotype of a Flower-Defective *Cosmos bipinnatus*

**DOI:** 10.3390/ijms20102503

**Published:** 2019-05-21

**Authors:** Fei Li, Wu Lan, Qin Zhou, Baojun Liu, Feng Chen, Sisi Zhang, Manzhu Bao, Guofeng Liu

**Affiliations:** 1 Key Laboratory of Horticultural Plant Biology, Ministry of Education; College of Horticulture and Forestry Sciences, Huazhong Agricultural University, Wuhan 430070, China; lifei8711@webmail.hzau.edu.cn (F.L.); w-lan@webmail.hzau.edu.cn (W.L.); zhouqin@webmail.hzau.edu.cn (Q.Z.); bjliumail@126.com (B.L.); chenf@webmail.hzau.edu.cn (F.C.); zhangsisi@webmail.hzau.edu.cn (S.Z.); mzbao@mail.hzau.edu.cn (M.B.); 2 Landscape plants research department, Wuhan Institute of Landscape Architecture, Wuhan 430081, China; 3 Guangzhou Institute of Forestry and Landscape Architecture, Guangzhou 510405, China

**Keywords:** Cosmos, floral mutation, *LEAFY UNUSUAL FLORAL ORGANS*, capitulum development

## Abstract

*LEAFY (LFY) and UNUSUAL FLORAL ORGANS (UFO)* homologous genes have been reported to play key roles in promoting the initiation of floral meristems in raceme- and cyme-type plants. Asteraceae, a large family of plants with more than 23,000 species, has a unique head-like inflorescence termed capitulum. Here, we report a floral defective plant of the garden cosmos named *green head* (*gh*), which shows homogeneous inflorescence, indistinguishable inflorescence periphery and center, and the replacement of flower meristems by indeterminate inflorescence meristems, coupled with iterative production of bract-like organs and higher order of inflorescences. A comparison of the *LFY*- and *UFO*-like genes (*CbFLY* and *CbUFO*) isolated from both the wild-type and *gh* cosmos revealed that *CbUFO* may play an important role in inflorescence differentiation into different structures and promotion of flower initiation, and the reduced expression of *CbUFO* in the *gh* cosmos could be associated with the phenotypes of the flower-defective plants. Further expression analysis indicated that *CbUFO* may promote the conversion of inflorescence meristem into floral meristem in early ray flower formation, but does not play a role in its later growth period.

## 1. Introduction

Flower initiation and development, two pivotal events during the life cycle of flowering plants, play a key role in the reproductive fitness of plants, as well as the successful production of crops. The branch bearing flowers of a plant are defined as the inflorescence, and the arrangement of flowers within the inflorescences is known as the inflorescence architecture [1,2]. During the evolution of angiosperms, various inflorescence architectures are produced in different species and they play an important role in plant reproductive adaptation and success [3,4]. Currently, except for a single flower, three basic architectural types of inflorescence have been proposed based on the activity of the inflorescence meristem in initiating lateral branches and the timing of its transition to floral meristems: raceme, cyme, and panicle [5,6]. Most inflorescences found in nature can be grouped into one of these three broad categories [7]. For instance, plants such as *Arabidopsis thaliana* and *Antirrhinum majus* develop the raceme-type inflorescences, with main inflorescence axes growing indefinitely and bearing flowers in lateral positions or lateral axes that reiterate this pattern. Petunia and tomato develop the cyme-type inflorescences, in which the main inflorescence meristems terminate in a flower after producing a new inflorescence meristem that reiterates this pattern. Unlike the raceme- or cyme-type inflorescences, the panicle-type inflorescence is extensively branched and is largely characteristic of grasses, such as oat and rice [6,7]. 

Extensive studies have been performed on the molecular regulation of inflorescence architecture and flower development for the three major inflorescence types. In the model plant *Arabidopsis*, an antagonistic interaction between *TFL1* (*TERMINAL FLOWER 1*) gene and flower meristem genes, such as *LFY* (*LEAFY*) and *AP1* (*APETALA1*), has been suggested to regulate the inflorescence architecture [8,9]. It has been reported that *TFL1* is specifically expressed in the center of inflorescence meristems to suppress the expression of *LFY* and *AP1* and prevent the meristems from gaining the floral identity, while *LFY* and *AP1* are strongly expressed in floral meristems to repress *TFL1* expression in this area and promote the floral identity [1,6]. *LFY* encodes a plant-specific transcription factor, with its orthologs present in most plant species as single-copy genes. Loss of function of *LFY* results in the conversion of flowers into inflorescence shoots, and the *ap1* mutation enhances the *lfy* mutant phenotype, indicating that *LFY* and *AP1* act synergistically to determine the floral meristem identity (FMI) [10]. In recent years, several genes have been identified to regulate the inflorescence and flower development in many other plant species, including *LFY* and *AP1* homologs, suggesting that the gene-regulatory networks controlling inflorescence and flower development are largely conserved among flowering plants. However, *pfo* (*proliferating floral organs*) mutant of *Lotus japonicas* [11] and *an* (*anantha*) mutant of tomato [12], which display flower-to-inflorescence conversion and cauliflower-like inflorescences, are other mutants of the *UFO* (*UNUSUAL FLORAL ORGANS*), aside from *LFY* or *AP1* orthologs [11,13]. UFO is an F-box protein that interacts with LFY to act as a transcriptional co-factor to regulate floral development in *Arabidopsis* [14]. The *ufo* mutants display defects in petal and stamen development [15,16]. However, analogous to *pfo* and *an* mutants, loss of function of the *UFO* ortholog in petunia *DOT* (*DOUBLE TOP*) leads to complete conversion of floral meristems into inflorescence meristems, indicating that *DOT*, as well as *PFO* and *AN*, is fully required to specify FMI [13]. The aforementioned reports indicate that *LFY/FLO* and *UFO/FIM* homologue genes play a key role in promoting the initiation of floral meristems in raceme- and cyme-type plants, respectively.

Asteraceae, a large family of plants with more than 23,000 species, has a unique head-like inflorescence, termed as capitulum, to distinguish itself clearly from other plants. The capitulum is a pseudanthium or false flower that resembles a solitary flower, but is actually comprised of numerous flowers [17,18,19]. The capitulum can be homogamous or heterogamous in Asteraceae, and all the flowers are similar in homogamous capitulum. However, in heterogamous capitulum there are obvious morphological differences between marginal flowers (ray flowers) and central flowers (disc flowers). The patterning of capitulum cannot be easily classified into the above three major inflorescence types, and the molecular mechanisms of Asteraceae capitula are not studied as extensively as those in the conventional model plants. Until now there have been different views on the origin of flower development of Compositae plants [17,20,21]. In recent years, Elomaa and his colleagues have identified several genes to regulate Asteraceae flower and inflorescence development, and they proposed that Asteraceae inflorescences resemble solitary flowers, not only morphologically, but also molecularly [18]. Several similar studies have been performed in some other species, such as sunflower (*Helianthus annuus*) and chrysanthemum (*Chrysanthemum* sp.) [22,23]. Most of these works are based on analysis of RNAi lines or identification of floral mutants, suggesting the importance of isolation of the floral mutant and its phenotype in advancing knowledge of capitulum development. 

Cosmos (*Cosmos bipinnatus*) is a popular ornamental plant in Asteraceae. The capitulum of cosmos is comprised of only one whorl of ray flowers at the periphery and about seven whorls of spiral-arrangement disc flowers. Recently, we have found that a floral defective plant of the garden cosmos displays a defect in flower meristem identity, resulting in reiterative initiation of inflorescences with bract-like organs rather than flowers in the capitulum. We named this plant *green head* (*gh*). Here, we report the morphological and ontogenetic characterization of the capitulum in the *gh* cosmos and compare the development process of inflorescence and flower development between the wild-type and flower-defective plants. Meanwhile, we tried to explore the potential cause for this mutation by cloning the *LFY* and *UFO* homologs and examining possible variations of their coding sequences and expression patterns in the *gh* cosmos. Our research confirms the role of *LFY* and *UFO* homologous genes in regulation of inflorescence and flower development previously reported in Asteraceae and enriches the information about the genetic regulation of flower development in Asteraceae. 

## 2. Results

### 2.1. Morphological and Ontogenetic Characteristics of Wild-Type and gh Cosmos

**Figure 1:**
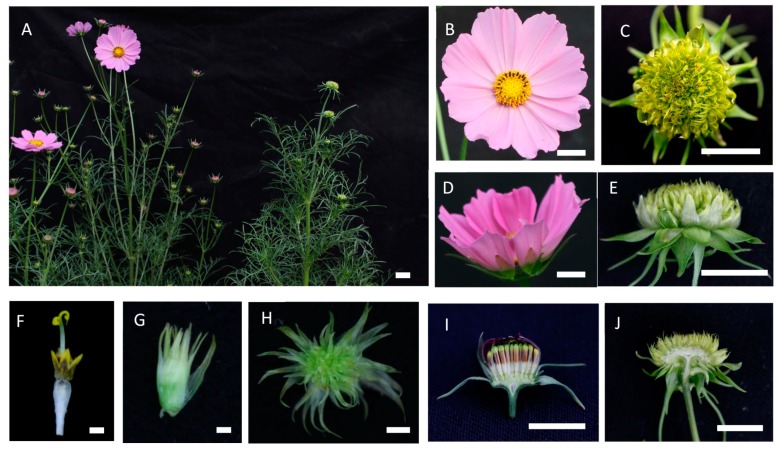
Morphological observation of wild-type and *gh* cosmos (**A**) Wild-type cosmos (left) and *gh* cosmos (right); (**B**,**D**,**I**) mature capitulum of wild-type cosmos; (**F**) disc flower of wild-type cosmos; (**C**,**E**,**J**) mature capitulum of *gh* cosmos; (**G**) separated tightly-folded structure is unwrapped in *gh* cosmos; (**H**) separated tightly-folded structure in *gh* cosmos. Scale bars of A–E, I, J = 1cm; scale bars of F, G, H = 1mm.

The wild-type cosmos has a typical heterogamous capitulum, which is surrounded by two whorls of involucral bracts (green leathery outer whorl and semilucent membranous inner whorl) and consists of two morphologically and functionally different types of flowers: a whorl of large and showy ray florets formed at the capitulum periphery, usually numbering eight for the single-flowered type of varieties, and about seven whorls of smaller disc florets at the center (Figure 1A,B,D). The marginal ray flowers are sterile with only a petal organ, and the central disk flowers are hermaphrodites with all four whorls of flower organs, including functional stamens and carpels (Figure 1B,D,F). The ray flowers develop next to the last series of involucral bracts (Figure 1D,I, and Figure S1D).

In contrast to the wild-type inflorescence, the capitulum of *gh* cosmos cannot form normal flowers, including the ray florets and disc florets. In the outer ring of the inflorescence, two whorls of leathery bracts and one whorl of membranous bracts can be seen to develop. Next to the last series of involucral bracts, we can see the whole inflorescence is homogeneous, the periphery and center of the inflorescence becomes indistinguishable, and no ray florets can be seen. In other words, the whole inflorescence structure becomes monotonous with a tightly folded structure emerging at the place where the normal ray or disc florets should grow (Figure 1C,E,J; Figure S1D,E,F). When the tightly folded structure is separated and unwrapped, it resembles a new head-like inflorescence in shape and structure with numerous ectopic bract-like organs stacking together (Figure 1G,H). Additionally, the number of leathery bracts was nearly twice as high in the flower-defective plant than in the wild type (Figure 1D,E, and Figure S2), indicating that the involucral bracts increased by one whorl in the flower-defective plant relative to the wild type.

**Figure 2:**
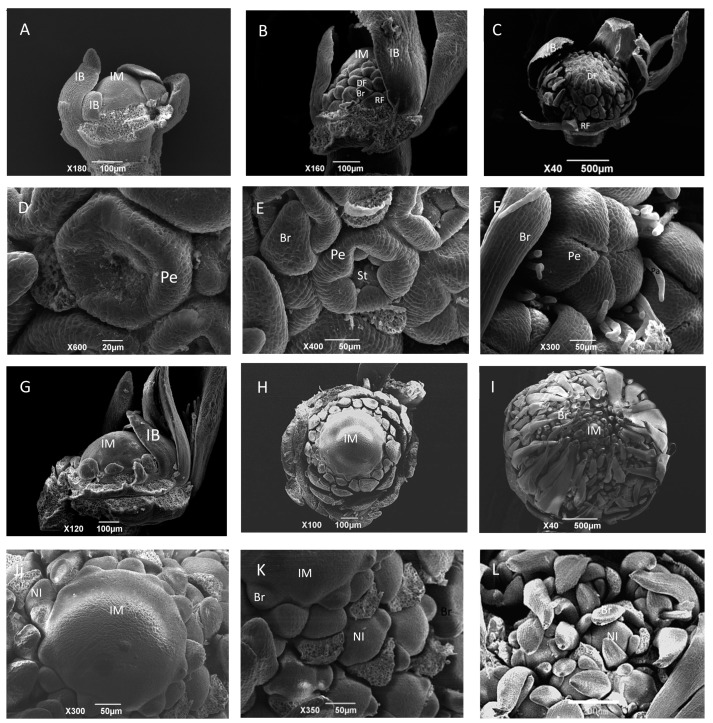
Anatomical observation of inflorescences in wild-type (**A**–**F**) and gh (**G**–**L**) cosmos with scanning electron microscopy (SEM). (**A**) A branch of inflorescence at the dome-shaped stage in wild-type cosmos; (**B**) the conversion of inflorescence meristems into flower meristems from bottom to apex in wild-type cosmos; (**C**) the apex of inflorescence meristem has already been converted into flower meristem in wild-type cosmos; (**D**) ring-shaped petal primordia were formed in a flower meristem of wild-type cosmos; (**E**) petal primordium was elongated and stamen primordium was formed in a flower meristem of wild-type cosmos; (**F**) the developed petals covered the stamen and carpel primordia, and the pappus bristles were formed in a flower meristem of wild-type cosmos. (**G**) A branch of inflorescence at the dome-shaped stage in gh cosmos; (**H**) the ectopic bracts emerged from the margins toward the apex in gh cosmos; (**I**) large numbers of new inflorescence meristems and ectopic bracts were formed; (**J**) the central part of the *gh* capitulum primordia remained undifferentiated; (**K**) new inflorescence meristems were formed at the base of ectopic bracts, and some ectopic bracts were removed; (**L**) the new inflorescence produced bracts at the periphery. IM, inflorescence meristem; IB, involucral bract; DF, disc flower meristem; RF, ray flower meristem; Br, bract; Pe, petal; St: stamen; Pa, pappus; NI, new inflorescence meristem. Scale bars = 20 μm in **D**; Scale bars = 50 μm in **E**, **F**, **J**, **K**; Scale bars = 100 μm in **A**, **B**, **H**, **G**; Scale bars = 200 μm in **L**; Scale bars = 500 μm in **C** and **I**.

**Figure 3:**
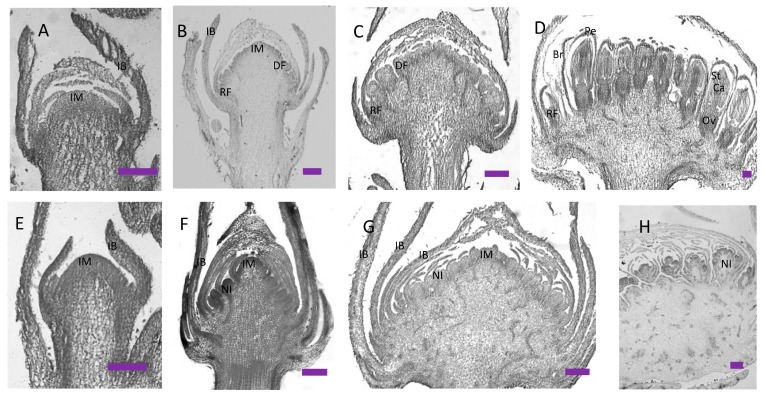
Histological staining of wild-type cosmos (**A**–**D**) and *gh* cosmos (**E**–**H**). (**A**) Young inflorescence meristem in wild-type cosmos; (**B**) the inflorescence meristem transforming into flower meristem in wild-type cosmos; (**C**) most flower meristem was formed in wild-type cosmos. (**D**) flower organs were formed in wild-type cosmos. (**E**) Young inflorescence meristem in *gh* cosmos; (**F**) ectopic bracts and new inflorescence meristems were formed in *gh* cosmos; (**G**) more new inflorescence meristems were formed in *gh* cosmos; (**H**) the inflorescence meristems produced the secondary inflorescence meristems in *gh* cosmos. IM, inflorescence meristem; IB, Involucral bract; DF, disc flower meristem; RF, ray flower meristem; Br, bract; Pe, petal; St, stamen; Ca, carpel; Ov, ovary; scale bars = 200 µm.

Based on scanning electron microscopy (SEM) and histological section analysis, the early inflorescence development of the wild-type cosmos can be divided into two different stages: the inflorescence primordia development stage and flower primordia differentiation stage. During the inflorescence primordia development stage, the whole inflorescence is approximately 0.2–1 mm in diameter, and little difference can be found between the wild-type cosmos and the *gh* cosmos at this stage, with their inflorescence primordia both emerging as a dome-shaped protuberance (Figure 2A,G; Figure 3A,E). At approximately 1 mm in diameter, the inflorescence of the wild-type cosmos enters the primordia differentiation stage, with the conversion of the inflorescence primordia from a whole structure into separate individuals (Figure 2B,C). The disc flower primordia were produced from the bottom to the apex (Figure 2B,C). At the abaxial base of each disc flower primordium, a bract primordium was also produced (Figure 2B and Figure 3B). The individual disc flower primordia are subsequently differentiated into the distinguishable structure of floral organs, which can be divided into several steps. First, the flower primordia develop from undifferentiated bumps to special cup-like structures (Figure 2B,C), followed by the initial formation of the ring-shaped petal primordia, and the differentiation of stamen and carpel primordia (Figure 2D,E). When the developed petals begin to cover the stamen and carpel primordia, the elongated pappi (whorl 1 organs) can be easily seen from the outside (Figure 2F). The ray flower primordia were defined at an early time next to the last series of involucral bracts, but they developed very slowly and eventually wrapped up all the mature disc flowers (Figure 1I, Figure 2B,C and Figure S1B,C). Compared to the wild-type inflorescence, notable differences can be observed in the capitulum of the *gh* plants at the flower primordia initiation and development stages after the differentiation of involucral bracts. However, the *gh* capitulum produce small bulges other than flower primordia from the margins to the apex in the capitulum (Figure 2H,I). These small bulges subsequently become flattened, fold toward the center of inflorescence and develop into bract-like organs (Figure 2H, Figure 3F and Figure S3), followed by the emergence of new bulges at the axils of these bract-like organs that will develop into secondary inflorescence meristems. The secondary inflorescence meristems never develop into cup-like or ring-like floral meristems, but produce more bract-like organs and new inflorescence meristems (Figure 2K,L; Figure 3G,H). With the emergence of more bract-like organs, the central undifferentiated region of the capitulum diminishes gradually, but the *gh* plant capitulum will maintain the undifferentiated status in the central part for a longer time than the wild type (Figure 2I,J; Figure S3). In the whole capitulum development process of the *gh* cosmos, no disc flower or ray flower primordium has been formed, and the secondary inflorescence meristems can form bract-like organs and new inflorescence meristems repeatedly at the later development stage (Figure 3H). Then, more and more bract-like organs will be formed in the green head, which will be tightly folded together and cover the new forming inflorescence meristem (Figure 1E,F). This observation indicates that the fate of ray flowers and disc flowers has not been determined during the inflorescence development, and the transition of inflorescence meristem into floral meristem is inhibited in the *gh* cosmos. 

### 2.2. Sequence Analysis of LFY and UFO Orthologs in Wild-Type and gh Plants

**Figure 4:**
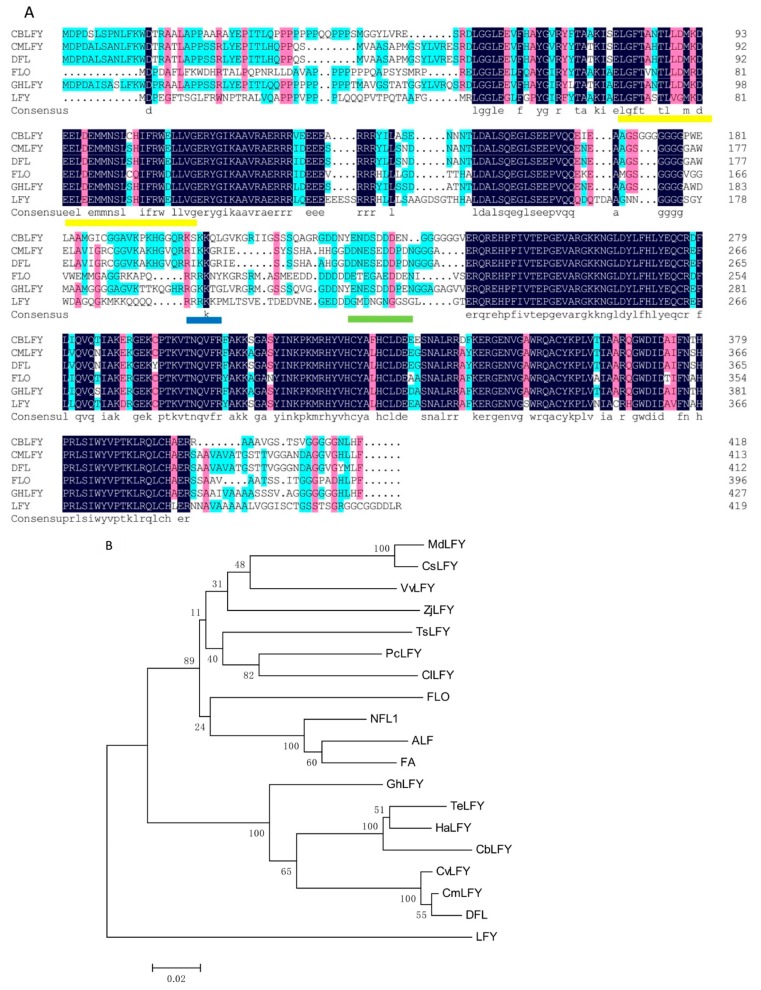
Multiple sequence alignment (**A**) and phylogenetic relationship (**B**) of CbLFY and LFY-like proteins. Identical amino acid residues in this alignment are shaded in blue; similar amino acid residues are shaded in red; the Leu zipper is indicated by a yellow underline; the acidic region is indicated by a blue underline; the basic region is indicated by a green underline; the sequences included in the analysis have the following accession numbers: CmLFY (Chrysanthemum × morifolium BAN19220.1), DFL (Chrysanthemum lavandulifolium AAT51708.1), FLO (Antirrhinum majus subsp. majus AAA62574.1), GhLFY (Gerbera hybrid cultivar ANS10152.1), LFY (Arabidopsis thaliana AED97525.1); (**B**) Phylogenetic relationship among FLORICAULA/LEAFY-like proteins. The protein sequences included in the analysis are TeLFY (Tagetes erecta AEG88962.2), CmLFY (Chrysanthemum × morifolium BAN19220.1), DFL (Chrysanthemum lavandulifolium AAT51708.1), CvLFY (Chrysanthemum vestitum AHY22453.1), HeLFY (Helianthus annuus XP_021984216.1), GhLFY (Gerbera hybrid cultivar ANS10152.1), CsLFY (Citrus sinensis AAR01229.1), PcLFY (Pistacia chinensis AGF33326.1), ClLFY (Clausena lansium ABF61861.2), VvLFY (Vitis vinifera AAM46141.1), TsLFY (Triadica sebifera ALO75821.1), MdLFY (Malus domestica NP_001280945.1), CsLFY (Chaenomeles sinensis BAD10959.1), ALF (Petunia × hybrida AAC49912.1), ZjLFY (Ziziphus jujuba NP_001310806.1), NFL1 (Nicotiana tabacum AAC48985.1), FA (Solanum lycopersicum AEO00262.1), LFY (Arabidopsis thaliana AED97525.1), and FLO (Antirrhinum majus subsp. majus AAA62574.1).

**Figure 5:**
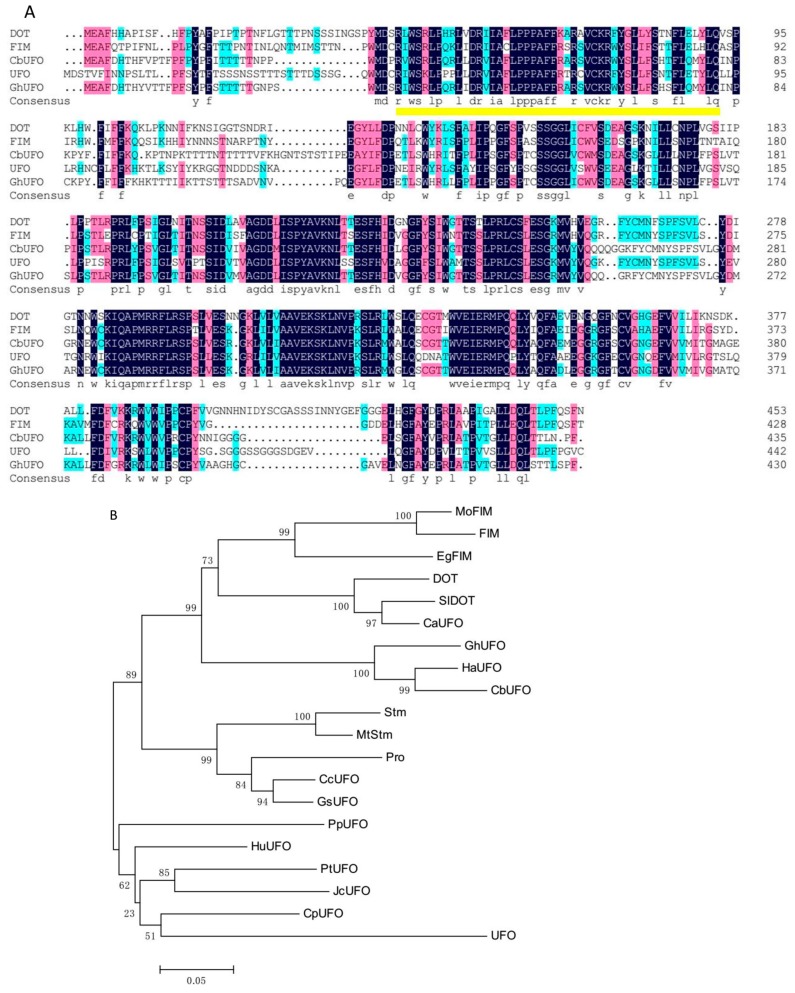
Multiple sequence alignment (**A**) and phylogenetic relationship (**B**) of CbUFO and UFO-like proteins. Identical amino acid residues in this alignment are shaded in blue, and similar amino acid residues are shaded in red. The F-box domain is indicated by a yellow underline. The protein sequences included in the analysis are GhUFO (Gerbera hybrid cultivar ANS10153.1), FIM (Antirrhinum majus bbm|341836), DOT (Petunia × hybrida ACA61781.1), UFO (Arabidopsis thaliana OAP18214.1). (**B**) Phylogenetic relationship among FIM/UFO-like proteins. The protein sequences included in the analysis are HaUFO (Helianthus annuus XP_021982608.1), GhUFO (Gerbera hybrid cultivar ANS10153.1), MoFIM (Misopates orontium CAJ44131.1), FIM (Antirrhinum majus AAB31352.1), HuUFO (Herrania umbratica XP_021300289.1), SlDOT (Solanum lycopersicum ACA61782.1), DOT (Petunia × hybrida ACA61781.1), CpUFO (Carica papaya XP_021891471.1), CcUFO (Cajanus cajan XP_020232435.1), CaUFO (Capsicum annuum NP_001311737.1), JcUFO (Jatropha curcas XP_012078537.1), EgFIM (Erythranthe guttata AAS46000.1), PpUFO (Prunus persica XP_007213358.1), GsUFO (Glycine soja KHN25935.1), Pro (Lotus japonicus AAN87351.1), PtUFO (Populus tomentosa AGW52144.1), Stm (Pisum sativum AAD01204.1), MtStm (Medicago truncatula AAX28871.1), and UFO (Arabidopsis thaliana OAP18214.1).

The orthologs of the key FMI genes *LFY* and *UFO* were cloned and identified from a wild-type cosmos plant, which were named as *CbLFY* (FLORICAULA/LEAFY like gene in *Cosmos bipinnatus*) and *CbUFO (UNUSUAL FLORAL ORGANS* like gene in *Cosmos bipinnatus)*, respectively. *CbLFY* had an open reading frame (ORF) sequence of 1254 bp and encoded a putative protein of 417 amino acids (Figure 4A), which is 57% identical to LFY, 65% identical to FLO, and 78% identical to CmLFY (FLORICAULA/LEAFY like protein in *Chrysanthemum x morifolium*). It also contains some specific regions, such as a putative short Leu zipper, a basic region formed by a core of Arg and Lys residues, and an acidic region, which can also be found in other angiosperm FLO/LFY proteins (Figure 4A). Phylogenetic tree analysis using the predicted protein sequences indicated that the CbLFY protein is closely related to HaLFY (Helianthus annuus) and TeLFY (Tagetes erecta), two LFY orthologs from Asteraceae species (Figure 4B), suggesting that *CbLFY* represents the *LFY* ortholog in cosmos. *CbUFO* had an ORF sequence of 1308 bp and encoded a putative protein of 435 amino acids containing F-box (Figure 5A), which is 60% identical to UFO and DOT, 62% identical to FIM, and 79% identical to GhUFO (*UNUSUAL FLORAL ORGANS* like protein in Gerbera hybrid cultivar). Phylogenetic tree analysis indicated that the CbUFO protein is clustered together with HaUFO (Helianthus annuus) and GhUFO, the orthologs of UFO from species in Asteraceae (Figure 5B), suggesting that *CbUFO* represents the *UFO* ortholog in cosmos.

To clarify the potential mutation of *CbLFY* and *CbUFO* genes in the *gh* plant, the coding sequences (CDS) of *CbLFY* and *CbUFO* were further amplified from 15 lines of wild-type cosmos and 3 individuals of the *gh* plant. Three different putative amino-acid sequences of CbLFY (named CbLFY-w1, CbLFY-w2, and CbLFY-w3) were obtained from the wild-type plants and one unique putative amino-acid sequence (named CbLFY-m) from the gh plants. A multiple alignment of these sequences revealed that the gene function might not be affected by the amino acid variation at the six sites in the wild-type CbLFY sequences (Figure S4A). The differences between CbLFY-m and CbLFY-w sequences are all located at these six variant sites, and the changed amino acid sequence of CbLFY-m is identical to at least one of the three CbLFY-w sequences, suggesting that CbLFY-m could have normal functions as the CbLFY-w in the wild-type cosmos. Similarly, three distinct putative amino-acid sequences of CbUFO (named CbUFO-w1, CbUFO-w2, and CbUFO-w3) were obtained from the wild-type cosmos and one putative amino-acid sequence (named CbUFO-m) from the *gh* cosmos. The multiple alignment analysis showed that the gene function could not be affected by the amino acid variation at the nine sites in the wild-type CbUFO sequences (Figure S4B). Compared to the wild-type CbUFO sequences, all the different amino acids of CbUFO-m are included in these sites and they are identical to the amino acids of at least one of the three CbLFY-w sequences, indicating that the *gh* phenotype is not derived from the CDS mutation of the *CbUFO* gene.

### 2.3. Expression of CbLFY and CbUFO in Wild-Type Cosmos

**Figure 6:**
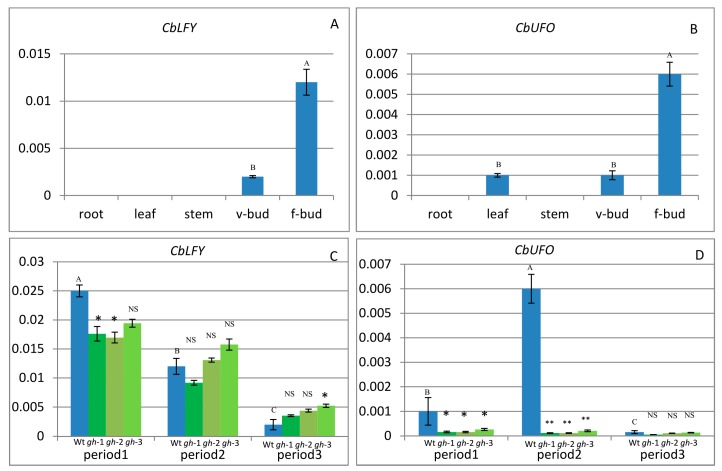
Expression of *CbLFY* and *CbUFO* in wild-type and *gh* cosmos. (**A**) Expression of *CbLFY* in different organs of wild-type cosmos; (**B**) expression of *CbUFO* in different organs of wild-type cosmos; (**C**) expression of *CbLFY* in different stages of wild-type cosmos (the left bar) and “green head” cosmos (the right three bars; gh-1, gh-2, gh-3); (**D**) expression of *CbUFO* in different stages of wild-type cosmos (the left bar) and “green head” cosmos (the right three bars; gh-1, gh-2, gh-3). Each value was the average of three different samples. Mean values with the same capital letter indicate no significant difference (*p* > 0.05). Each value represents the mean from three biological replicates; * indicates significantly different at 0.05 level; ** indicates significantly different at 0.01 level; NS indicates no significant difference (*p* > 0.05); v-bud = vegetable bud; f-bud = flower bud.

**Figure 7:**
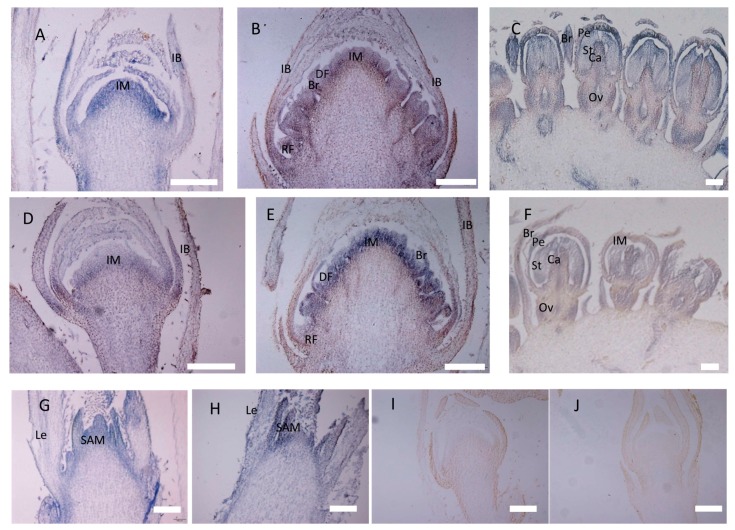
In situ hybridization of *CbLFY* and *CbUFO* in wile-type cosmos. (**A**) Expression of *CbLFY* was detected in the young dome-shaped IM and IB; (**B**) expression of *CbLFY* was detected in developed inflorescence meristem in transition to floral meristem, disc flower meristem, and young bract; (**C**) expression of *CbLFY* was detected in the young bract, petal, stamen, carpel, and ovary; (**D**) expression of *CbUFO* was detected in the young IM, but did not reach the surface and was absent from IB; (**E**) expression of *CbUFO* was detected in developed inflorescence meristem in transition to floral meristem, disc flower meristem, and young bract, but was absent in the ray floral meristem; (**F**) expression of *CbLFY* was detected in young petal, stamen, carpel, and ovary; (**G**) expression of *CbLFY* was detected in the vegetative shoot apical meristem (SAM), but was absent from the leaf; (**H**) expression of *CbUFO* was detected in the vegetative shoot apical meristem (SAM) and the leaf; (**I**) sense probe for *CbLFY*; (**J**) sense probe for *CbUFO*. SAM, shoot apical meristem; Le, leaf primordium; IM, inflorescence meristem; IB, involucral bract; DF, disc flower meristem; RF, ray flower meristem; Br, bract; Pe, petal; St, stamen primordium; Ca, carpel; Ov, ovary. Scale bars = 200 um.

The transcription levels of *CbLFY* and *CbUFO* in various cosmos tissues and at different capitulum development stages were investigated by quantitative reverse transcription (RT)-PCR (Realtime-Polymerase Chain Reaction). The results showed that the *CbLFY* expression was absent from roots, leaves, and stems, but was detectable in vegetative buds and strongly detectable in flower buds (Figure 6A and Figure 7G). Statistical analysis showed significant differences in expression between flower buds and vegetative buds. The *CbUFO* expression was not detectable in roots and stems, but weakly detectable in leaves and vegetative buds, and strongly detectable in flower buds (Figure 6B and Figure 7H). Statistical analysis showed significant differences in expression between flower buds and vegetative buds, and no significant differences between young leaves and vegetative buds. That could indicate some roles of *CbUFO* in vegetative organs. During capitulum development, the expressions of *CbLFY* and *CbUFO* were analyzed at three representative periods: Period 1 (P1), forming inflorescence meristem; Period 2 (P2), forming flower meristem; and Period 3 (P3), forming flower organs. *CbLFY* was most highly expressed at the early stage (P1), followed by a gradual decrease at the later stages (P2 and P3); *CbUFO*, however, showed the highest expression level at the middle stage (P2) (Figure 6C,D).

The expression domains of *CbLFY* and *CbUFO* were further investigated by in situ hybridization during the three inflorescence development stages. At the P1 stage, the *CbLFY* transcripts were uniformly and highly accumulated in the whole dome-shaped inflorescence meristem, and the expression was also detected in the basal part of the involucral bracts (Figure 6A and Figure 7A). However, the expression of *CbUFO* at this stage was only located in the region below the surface of the dome-shaped inflorescence meristem (Figure 7D). At the flower differentiation stage, the *CbLFY* transcripts were detectable in both early disc and ray flower meristems, but the signal became weaker in the later flower development stage (Figure 7B). When the flower organs were produced in the P3 stage, *CbLFY* was expressed in developing bracts, petals, stamens, and carpels (Figure 7C). *CbUFO* accumulated in similar regions as *CbLFY* during disc flower meristem differentiation and development stages, but showed a stronger signal than *CbLFY*. However, *CbUFO* was not expressed in the primordia of ray Flowers. After the formation of floral organs, a low *CbUFO* expression level was detected in petals, stamens, carpels, and ovaries (Figure 7E,F). No hybridization signal was obtained with the *CbLFY* and *CbUFO* sense RNA probe (Figure 7I,J). 

### 2.4. Expression of CbLFY and CbUFO in gh Cosmos

**Figure 8:**
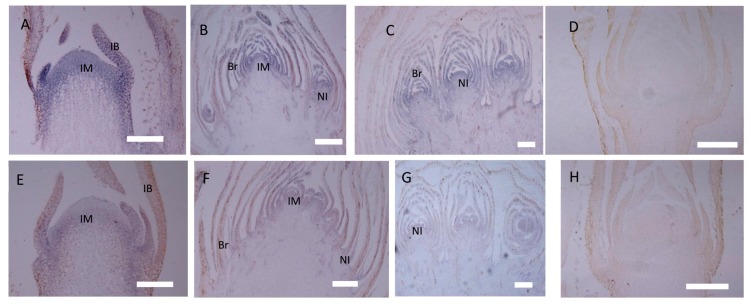
In situ hybridization of *CbLFY* and *CbUFO* in *gh* cosmos. (**A**) Expression of *CbLFY* was detected in the young dome-shaped IM, but did not reach the surface, and was absent in young IB; (**B**) expression of *CbLFY* was detected in IM, bract, and new forming inflorescence; (**C**) expression of *CbLFY* was detected in bract and new forming inflorescence of the *gh* cosmos; (**E**) expression of *CbUFO* was detected in the young IM, but did not reach the surface and was absent from the IB; (**F**) expression of *CbUFO* was detected in IM, young bract, and new forming inflorescence; (**G**) expression of *CbUFO* was detected in young bract and new forming inflorescence; (**D**) sense probe for *CbLFY*; (**H**) sense probe for *CbUFO*. IM, inflorescence meristem; IB, involucral bract; Br, bract; NI, new inflorescence bars = 200 µm.

In the *gh* cosmos, *CbLFY* was also most highly expressed at the early inflorescence development stage (P1), and the *CbLFY* expression was slightly lower in the *gh* cosmos than in the wild type, with a significant difference (*p* < 0.05) in two lines of *gh* and no significant difference in one line (Figure 6C). At P2 and P3 stage, the *CbLFY* expression was slightly higher in the *gh* than in the wild type, with only one line of *gh* showing a significant difference (*p* < 0.05) from the wild type at P3 stage (Figure 6C). However, the expression of *CbUFO* remained at a low level during the three periods (Figure 6D). At both P1 and P2 stages, all three lines of *gh* showed a significant difference (*p* < 0.01) from the wild type, especially at the P2 stage when flower meristem was being formed in the wild-type plants, but no significant difference was observed at P3 stage (Figure 6C,D). In situ hybridization analysis demonstrated that the *CbLFY* was expressed at the same domain and had a similar expression level to that of the wild-type plants at P1 stage (Figure 8A), while *CbUFO* showed a low expression level below the surface of the dome-shaped IM at this stage (Figure 8E). During P2 and P3 stages, *CbLFY* and *CbUFO* were expressed in the top inflorescence meristem and new forming inflorescence meristems. Meanwhile, *CbLFY* was also expressed in the emerging bract primordia, while *CbUFO* was only weakly expressed (Figure 8B,C,F,G). No hybridization signal was obtained with the *CbLFY* and *CbUFO* sense RNA probe (Figure 8D,H).

### 2.5. Transgenic Analysis

**Figure 9:**
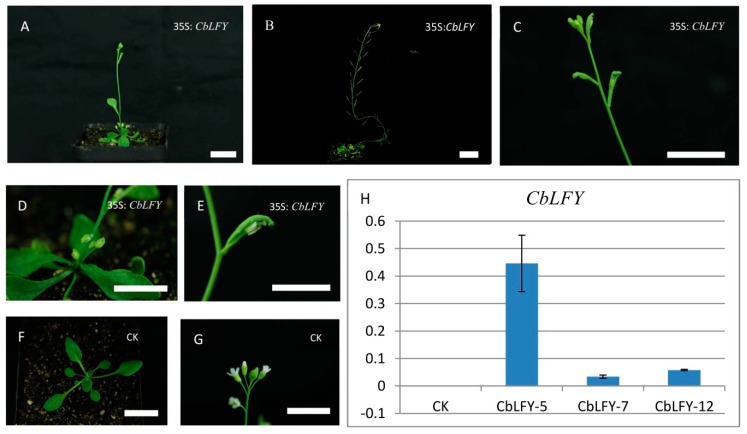
Phenotypes of *35S:CbLFY* transgenic *Arabidopsis* T_2_ lines and expression analysis of the transgene. (**A**) Early flowering phenotype of the transgenic line; (**B**) transgenic plants in fruit period; (**C**) lateral inflorescence turns into flowers; (**D**) the transgenic line produces more sprouts from the base and lateral branches develop into flowers; (**E**) the Cauline leaves are curved to wrap the flowers, and the pistils become longer and protrude outside petals in the flowers that are not yet blooming; (**F**,**G**) phenotype of group CK (Blank control); (**H**) expression analysis of the transgene. The scale is 2 cm.

**Figure 10:**
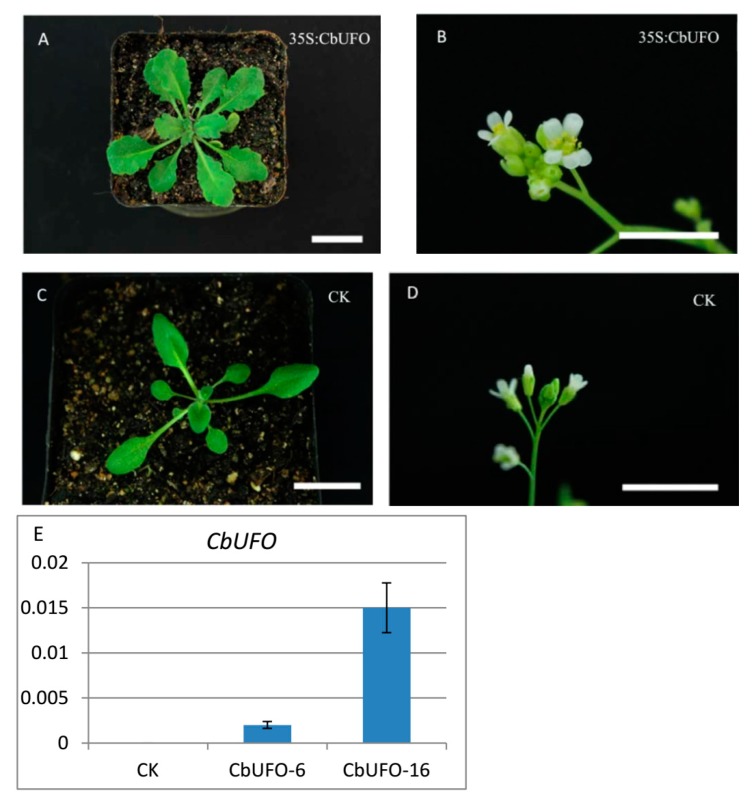
Phenotypes of *35S:CbUFO* transgenic *Arabidopsis* T_2_ lines and expression analysis of the transgene. (**A**) Ectopic expression of CbUFO resulting in serrated leaves; (**B**) ectopic expression of CbUFO led to the production of flowers with extra petals and stamens; (**C**,**D**) phenotype of group CK; (**E**) expression analysis of the transgene. The scale is 2 cm.

In order to identify the gene functions of *CbLFY* and *CbUFO*, we created the overexpression constructs *35S*:*CbLFY* and *35S*:*CbUFO*, and transferred them separately into *Arabidopsis* (due to lack of genetic transformation system in cosmos). Similar to the phenotypes of over-expressed *LFY* and *UFO* in *Arabidopsis* [10,15,24], the *CbLFY* transgenic lines have different degrees of early flowering phenotypes and the lateral shoots and inflorescences all turned into flowers in the transgenic lines with a severe phenotype (Figure 9; Table S1). The *CbUFO* transgenic lines also exhibited the phenotypes of increased petal and stamens and serrated leaves, but not early flowering (Figure 10; Table S1). These observations indicated that the *CbLFY* and *CbUFO* have the functions of conserved functions as their counterparts in *Arabidopsis*.

## 3. Discussion

### 3.1. Potential Causes for the gh Mutation

As cosmos is a strictly self-incompatible species, and the gh cosmos is infertile in a natural state, it could have originated from the variation of heterozygous wild-type plants in the same area. In the past several years, we have collected the seeds from potential heterozygotes. Despite a low probability of obtaining cosmos material from these seeds, we have been able to obtain several phenotypically stable cosmos lines each year, but we cannot clearly determine which individual is the heterozygote. Considering the lower probability of simultaneous mutation of multiple genes under natural conditions, we infer that the *gh* cosmos was derived from the mutations of a single or several recessive genes, and more likely from a single gene mutation.

The *gh* phenotype shows the mutation of flower structure into inflorescence structure, which is very similar to the mutation of *LFY* and *UFO* homologous genes in some species, such as *flo* of snapdragon [25], *an* of tomato [12], *alf* and *dot* in petunia [13,26], and *pfo* in *Lotus japonicus* [11]. In Asteraceae plants, the phenotype of the *gh* showed the loss of floral organ identities and repetitive initiation of inflorescence primordia, with the secondary and tertiary inflorescence primordia subtended by bract-like structures, which is especially similar to the phenotype of severe RNAi transgenic lines of *GhLFY* and *GhUFO* and the *GhUFO*-lacking mutant “PingPong” described in Gerbera [18]. Given that the role of *LFY* and *UFO* homologous genes in flower development regulation have been confirmed in most angiosperms, such as Asteraceae plants, and that the *gh* phenotype is not derived from the CDS mutation of the *CbLFY* or *CbUFO* gene, the decrease of *CbUFO* expression can be assumed to be responsible for the absence of the floral organ phenotype in cosmos. Although expression analysis showed that *CbLFY* was slightly down-regulated in two *gh* plants in P1 stage (Figure 6C), there was no significant difference between the expression of the third *gh* plant and wild-type plants (Figure 6C), and this slight difference could also exist in other wild-type plants, so we infer that the expression of *CbLFY* gene did not change significantly in *gh* plants. The changes in the expression of *CbUFO* can be attributed to mutations in the promoter regions, due to the effect of DNA methylation or mutations in upstream regulatory factors. However, the *gh* cosmos in our research can produce more whorls of involucral bracts than the wild-type plants, which differed from the *GhUFO*-lacking mutant “PingPong” in Gerbera and RNAi lines of *GhLFY* or *GhUFO*. It has been reported that bract suppression and meristem function in controlling floral patterning might be co-regulated in many dicotyledonous plants [27,28,29]. *UFO* in Arabidopsis has also been reported to play a role in bract suppression [29]. Therefore, the increase of involucral bracts is possibly due to the decrease of *CbUFO* expression. However, there are no reports available about suppression of involucral bracts by *LFY* or *UFO* homologous genes. Even in the RNAi lines of *GhLFY* or *GhUFO* in Gerbera, no increase was observed in the involucral bracts, and the morphological changes for the increase of involucral bracts occurred before inflorescence development. The mechanism for the development of involucral bracts may be different from that of single flower bracts, suggesting that the increase in the involucral bracts may also be caused by some other mutations. Thus, it could be deduced that some mutations from upstream regulators led to an increase in involucral bracts and a decrease in *CbUFO* expression. This hypothesis is more plausible for the relatively low probability of simultaneous mutation of multiple genes in a natural state. In *Arabidopsis*, *SOC1*, *AGL24*, and *SPL3* are proposed as direct upstream activators of *LFY* [30,31], but upstream transcription factors of *UFO* homologous genes have been seldom reported, which may be worth further study in the future.

### 3.2. Functional Evolution of LFY- and UFO-like Genes in Asteraceae

In previous studies, *LFY* and *UFO* homologous genes can both regulate the transition from inflorescence meristem to floral meristem in different Angiosperms [8,28,29]. In the present research, *CbLFY* and *CbUFO* have been confirmed to have the functions of conserved proteins as their counterparts in *Arabidopsis*. *CbLFY* was most highly expressed at the early stage of inflorescence development, indicating that it plays a very important role in the inflorescence development. During floral development, *CbLFY* can be detected both in ray flowers and disc flowers, indicating *CbLFY* can regulate the development of both ray flowers and disc flowers in cosmos. Our research partly confirmed the conclusion in gerbera that *GhLFY* has evolved a novel role in defining the IM determinacy and the conserved functions for development of individual flowers. However, *CbLFY* is expressed not only in reproductive meristems, but also in vegetative meristem, which is obviously different from the expression pattern of *GhLFY* in Gerbera. In some other plants, *LFY* homologous genes were also reported to be expressed in the vegetative buds, such as the *DFL* in Dendranthema lavandulifolium, *VcLFY* in violet cress, *NFL* in tobacco, and *FA* in tomato [30,31,32], although the role of *LFY* in the vegetative meristematic region is still unclear. This result is different from two previous studies on Arabidopsis and snapdragon, which showed the *LFY/FLO* gene was not expressed in the vegetative tissues [5,17]. The variation in the expression pattern of *LFY* homologs in different Asteraceae species suggests the existence of functional divergence for this gene in Asteraceae. 

According to previous studies, the spatial-temporal expression patterns of *UFO* homologs showed differences in cymose and racemose plants. In the typical racemose plant *Arabidopsis*, *UFO* is expressed in the apical meristem throughout the vegetative phase [10], while the *UFO* ortholog *DOT* in petunia, a typical cymose plant, is inactive during the vegetative phase and only expressed in the inflorescence meristem and the flower meristem [8]. *UFO* homologs play a major role in regulating the cymose inflorescence meristem pattern, but appear to perform a minor role in conferring floral meristem identity in racemose species [33]. In a previous study, *GhUFO* was proved as the master regulator of flower meristem identity in gerbera [18]. In our study, the expression of *CbUFO* was detectable by the naked IM at the early inflorescence development stage (P1), implying *CbUFO* can also play a role in the early development of inflorescence. This is different from the gerbera research reporting that *GhUFO* was not expressed in the naked IM [18]. Additionally, the expression of *CbUFO* reached the highest level at the P2 stage when the flower meristem was forming, confirming that it may play a more important role in controlling the floral meristem identity as the master regulator of FMI in cosmos, with the same function in cymose species and gerbera [33]. However, the in situ hybridization results showed that *CbUFO* expression was only detected in disc flowers, but not in developed ray flower, indicating that *CbUFO* gene may play a different role in regulating disc and ray flowers. A further look at the process for determining the fate of ray flowers showed that *CbUFO* is strongly expressed in the early stage of determining ray flower primordia, followed by a weak expression and finally the absence of *CbUFO* when the ray flower primordia are totally determined (Figure S5A–D). This indicated that *CbUFO* may promote the conversion of inflorescence meristem into the floral meristem in ray flower, but does not play a role in its later development period. It has also proved that *GhUFO* plays a more pronounced role in the patterning of disc flowers in gerbera. In the present study, *CbUFO* is also confirmed to be expressed in the vegetative buds and young leaf organs, which is opposite to the results in gerbera, but more similar to racemose species, such as *Arabidopsis*. However, the functions of *UFO*-like genes are very unclear in vegetative buds. We failed to detect any morphological differences in the phenotypes of vegetative buds in wild-type and *gh* plants (Figure S6). Although *UFO* gene is expressed in the vegetative buds of *Arabidopsis*, the RNAi lines of *UFO* gene showed no distinct phenotype in *Arabidopsis* [15,29]. This could imply that *UFO-like* genes may play a redundant regulatory role during vegetative growth. 

Currently, we still cannot confirm whether the capitulum inflorescence in Asteraceae plants belongs to raceme or cyme inflorescence based only on the expression pattern of *CbLFY* and *CbUFO*. However, the bracts could represent key architectural markers [32], and the capitulum inflorescence could be regarded as determinate (with a definite boundary and terminating in a flower) in the plant. Even in the floral defective plants of cosmos, the gh inflorescence exists as a deterministic structure throughout the plant. Therefore, the indeterminate internal unit structure of inflorescence in the *gh* (the indefinite proliferation of inner *gh* inflorescence to produce new inflorescence primordia with no terminating flower) might be caused by the decreased expression of *UFO-like* gene.

## 4. Methods and Materials

### 4.1. Plant Material

The wild-type (WT) and flower-defective (*gh*) plants of *Cosmos bipinnatus* used in this experiment were naturally collected from the field at the experimental station of the College of Horticulture and Forestry Sciences, Huazhong Agricultural University, Wuhan, China. The plants were grown under natural conditions using standard agricultural practices. The *gh* cosmos was obtained from the natural mutation of the WT cosmos. They did not grow closely together, but in the same area and the same environment. We collected the seeds of wild-type plants nearby, hoping to obtain *gh* plants from these seeds. We also preserved the mutant plants by asexual propagation, such as cutting and tissue culture.

### 4.2. Stereomicroscope Observation 

Flower buds (0.5-4 mm) of the wild-type and *gh* cosmos at the early developmental stages were observed under a stereomicroscope (SZX16, OLYMPUS, Japan). The outer bracts of the capitulum were removed to expose the central inflorescence meristem and the developing flower primordia when needed.

### 4.3. Histological Sections

Samples of young capitulum at different developmental stages (0.5-4 mm) for histological sections were collected and fixed in FAA (Formalin–acetic acid–alcohol, 50% ethanol, 5% acetic acid, and 5% formaldehyde) at room temperature for 2 days, followed by embedding in paraffin after treatment with different concentrations of alcohol and xylene. The paraffin-embedded samples were cut in 8 µm (RM2265, Leica Biosystems, Wetzlar, Germany), then examined and photographed using the BX53 Microscope (OLYMPUS) equipped with a U-HSCBM (OLYMPUS) digital camera.

### 4.4. Scanning Electron Microscopy

For SEM analysis, a time series of the capitula (0.5-4 mm) of the wild type and *gh* cosmos were collected at early developmental stages and hand dissected under a stereomicroscope. When necessary, excessive involucral bracts were removed to expose the central inflorescence meristem and the developing flower primordia. The samples were first stored in 2.5% glutaraldehyde for 24 hours at 4 °C, followed by rinsing in 0.1 mol/L phosphate buffer (pH7.5) and dehydration by alcohol (10%, 20%, 30%, 40%, 50%, 60%, 70%, 80%, 90%, and 100%) in turn. Finally, the samples were dried at the critical point of Autosampler 810 and plated with gold in a Hummer V sputter coater. The treated samples were examined by SEM (JSM-6390LV, JEOL Ltd., Tokyo, Japan). The obtained SEM images were further edited using Adobe Photoshop CS5.

### 4.5. Nucleic Acid Isolation

Total RNA was extracted from the young capitula at different developmental stages using Total RNA Kit (Aidlab Biotechnologies Ltd., Beijing, China). First-strand cDNA was synthesized using cDNA synthesis (Takara Bio Inc., Shiga, Japan). Genomic DNA was extracted from different developmental stages of the apical bud by the modified CTAB-based method [33].

### 4.6. Cloning of CbLFY and CbUFO

Gene-special primers were designed based on the conserved regions of *LFY* and *UFO* homologs. The conserved fragments of *CbLFY* and *CbUFO* were amplified with cDNA synthesized from the young capitula mRNA, and the full-length cDNA sequences of *CbLFY* and *CbUFO* genes were obtained using 3′-RACE and TAIL-PCR [34]. The primers used for all the experiments are shown in Table S2. Nucleic acid sequences and amino acid sequences were shown in Table S3.

### 4.7. Phylogenetic Analysis

The sequences of LFY-like and UFO-like proteins were retrieved from GenBank (https://blast.ncbi.nlm.nih.gov/) and aligned using ClustalW program in MEGA 5.0 (MEGA software development team, Tempe, AZ, USA). The phylogenetic trees of the LFY-like and UFO-like proteins were constructed using MEGA 5.0 with the Neighbor-Joining (NJ) method with 1000 bootstrap replicates. 

### 4.8. The qPCR Analysis

Real-time quantitative RT-PCR was performed to measure the *CbLFY* and *CbUFO* expressions in different organs and different capitulum developmental stages of the wild-type and *gh* cosmos. Total RNA was extracted from young roots, young stems, and young leaves of one-month seedlings. Capitula were sampled from the wild-type and *gh* cosmos at three periods for RNA extraction and real-time quantitative RT-PCR: period 1 (P1), capitula with a diameter of 0-1 mm that were forming inflorescence meristems; period 2 (P2), capitula with a diameter of 1-2 mm that were forming flower meristems and ectopic bracts for the wild type and *gh* plants, respectively; period 3 (P3), capitula with a diameter of 2-4 mm that were forming flower organs and secondary inflorescence meristems in wild-type and *gh* plants, respectively. RNA samples for the qPCR analysis were collected from three independent plants of the wild-type and *gh* (*gh-1, gh-2, gh-3*), respectively. *CbEF1α*, an *EF1α* homolog gene in cosmos, which was cloned by RT-PCR and confirmed by sequence-blast with homologous genes in NCBI, was selected for data normalization and calculation of the relative mRNA levels. The real-time quantitative RT-PCR was performed with the SYBR Premix Ex Taq (Takara, Japan) and the ABI7500 Real-Time System (Applied Biosystems, Carlsbad, CA, USA). The reaction was carried out in a 10 μl RT-reaction mixture containing 5 μL 2× SYBR Green Master Mix (TAKARA), 0.2 μL forward and reverse primers (10 μmol/μL), and water to a final volume of 10 μL, under the following cycling conditions: an initial denaturing step of 10 min at 95 °C, followed by 40 cycles of 5 s at 95 °C, and 34 s at 60 °C. All qPCR experiments were carried out in three biological replicates. All the data were analyzed using the 2^−ΔΔ*C*T^ method [35]. All the primers were designed by Primer-BLAST online (http://www.ncbi. nlm.nih.gov/tools/primer-blast/) and shown in Table S2.

### 4.9. In Situ Hybridization

A time series of capitula of both the wild-type and *gh* cosmos were collected at early developmental stages for in situ expression analysis. Tissues were fixed by dehydration in an increasing gradient series of ethanol, clearing with xylene, and embedding in Paraffin [36,37]. The sections were cut in 8 µm (RM2265, Leica Biosystems, Germany). 

Gene specific probes were synthesized for *CbLFY* (307 bp) and *CbUFO*(300 bp) according to Braissant and Wahli, 1998 [38], using a PCR-amplified fragment with a T7 promoter sequence appended to the 5’ end and labeled following the instructions of the DIG RNA labelling Kit (Roche, Basel, Switzerland). Hybridization and detection were carried out as described by Neta et al. [39]. Sections were examined and photographed using the BX53 Microscope (OLYMPUS) equipped with a U-HSCBM (OLYMPUS) digital camera.

### 4.10. Transgenic Analysis

The full-length cDNA sequences of the *CbLFY* and *CbUFO* genes were amplified and cloned into pMD-18T vector, respectively (Takara, Japan). For genetic transformation, we used the modified *pmv* vector to create the overexpression constructs 35S:*CbLFY* and 35S:*CbUFO*. *Agrobacterium tumefaciens* GV3101 containing the respective expression vectors was used for *Arabidopsis* (Col-0) transformation via the floral dip method [40]. The transformed seeds were surface-sterilized separately with 8% (*v*/*v*) sodium hypochlorite solution followed by 95% (*v*/*v*) ethanol. Murashige and Skoog (1/2MS) medium supplemented with 50 mg/L kanamycin sulfate (Biosharp, Beijing, China) and 50 mg/L cefotaxime sodium salt (Biosharp, China) was used to screen transformants. Then, the plants that survived were transplanted into soil and grown under long-day conditions (16 h light/8 h dark) at 22 °C to obtain self-pollinated seeds. The transgenic plants of the T2 generation lines, which fitted a segregation ratio of 3:1, were chosen to record flowering time and floral phenotype. Seedlings were grown in a growth incubator at 22 °C under long day (LD) conditions (16/8h, light/dark). Flowering time and the number of rosette leaves were measured when the plants bore a 1 cm long inflorescence. Three transgenic lines of *CbLFY* and two transgenic lines of *CbUFO* were chosen for analysis of the expression by real-time quantitative RT-PCR. Arabidopsis *EF1α* gene was used as the reference gene to normalize small differences in template amounts. The expression analysis was carried out using 14-d-old seedlings.

### 4.11. Data Analysis

All data analyses were performed using SPSS statistical software (SPSS, Chicago, IL, USA). Values were expressed as the mean ± standard deviation (SD) of three replications. Statistical significances of the differences between various groups were analyzed using the Fisher’s least significant difference (LSD) procedure. For comparison between two groups, Student’s t-test was used. A p-value of < 0.05 and <0.01 was considered significant and highly significant, respectively.

## 5. Conclusions

In this study, a floral defective plant in cosmos was identified to show three major characteristics in contrast to the wild type: (i) its capitulum cannot form normal florets, but produces a “green flower head” (named as *gh*); (ii) the *gh* capitulum produces more whorls of green involucral bracts and remains indeterminate for a longer time than the wild type; (iii) the whole inflorescence becomes homogeneous, the periphery and center of the inflorescence become indistinguishable, and its flower meristem is replaced by an indeterminate inflorescence meristem, leading to iterative production of bract-like organs and higher order of inflorescences. Given that *CbLFY* and *CbUFO* have the functions of conserved proteins as their counterparts in *Arabidopsis*, and the *gh* phenotype is not derived from the CDS mutation of the *CbLFY* or *CbUFO* genes, the *gh* phenotype could be associated with the reduced expression of *CbUFO* in the *gh* cosmos. Expression analysis of both wild-type and *gh* cosmos indicated that *CbLFY* was more involved in promoting the early inflorescence development, while *CbUFO* was more involved in inflorescence differentiation into different structures and promoting flower initiation. A further analysis of the expression of the *CbUFO* gene in early ray flower formation revealed that the *CbUFO* gene may play a different role in regulating disc and ray flowers. *CbUFO* is strongly expressed in the early stage of determining ray flower primordia, followed by a weak expression and finally the absence of its expression when the primordia of ray flowers are totally determined. The overall results indicated that *CbUFO* may promote the conversion of inflorescence meristem into floral meristem in ray flowers, but does not play a role in its later growth period.

## Supplementary Materials

Supplementary materials can be found at https://www.mdpi.com/1422-0067/20/10/2503/s1.

## Abbreviations

*UFO*	*unusual floral organs*
*LFY*	*leafy*
*TFL1*	*terminal flower 1*
*AP1*	*apetala1*
*DOT*	*double middle*
IM	inflorescence meristem
FM	floral meristem
FUM	floral unit meristem
FMI	floral meristem identity
RT-PCR	reverse transcription PCR
qPCR	real-time quantitative RT-PCR
