# Supplementary material for: Reduced Expression of CbUFO Is Associated with the Phenotype of a Flower-Defective Cosmos bipinnatus
Source: Int J Mol Sci. 2019 May 21;20(10):2503. doi: 10.3390/ijms20102503 (PMC6566773; doi:10.3390/ijms20102503)
Supplement: Supplementary file 1 [file ijms-20-02503-s001.zip › supplementary files/Fig S1.docx]

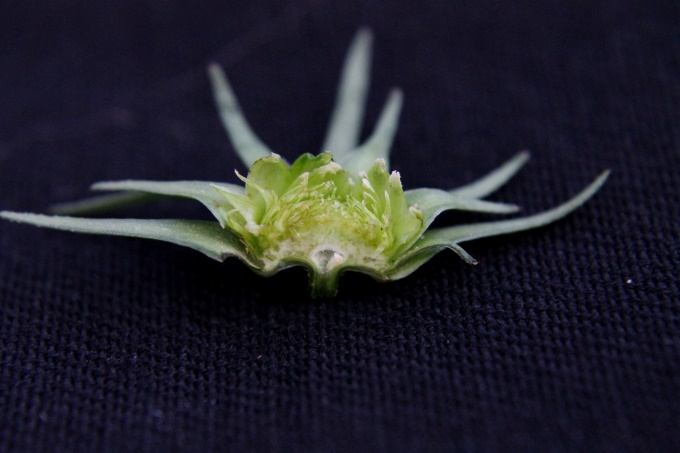

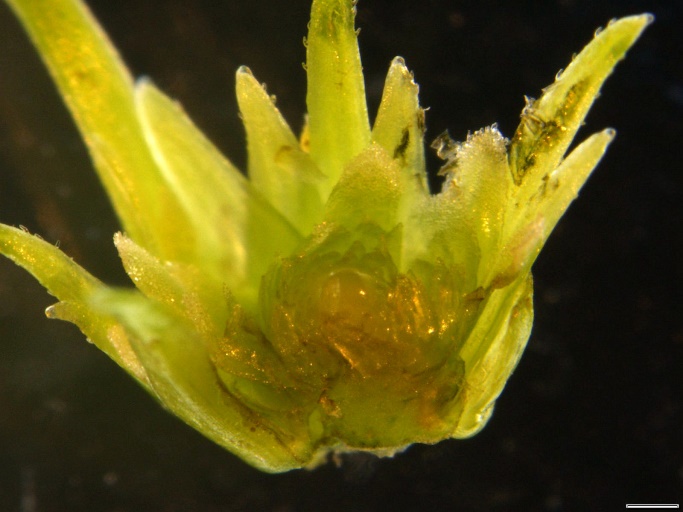

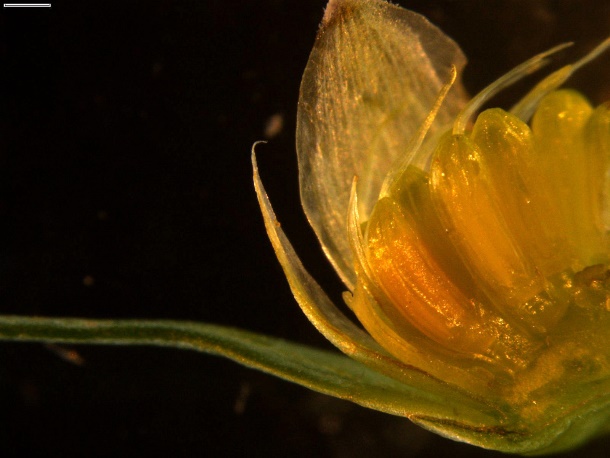

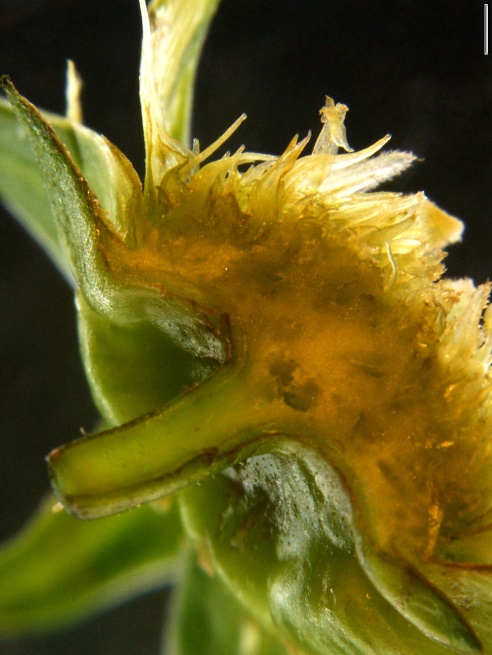

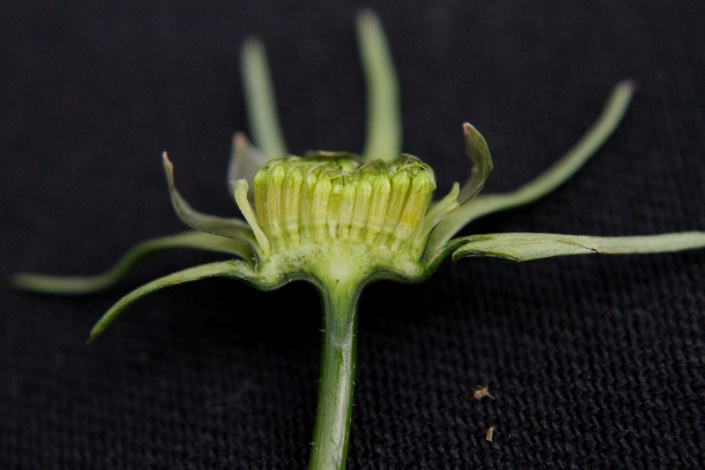

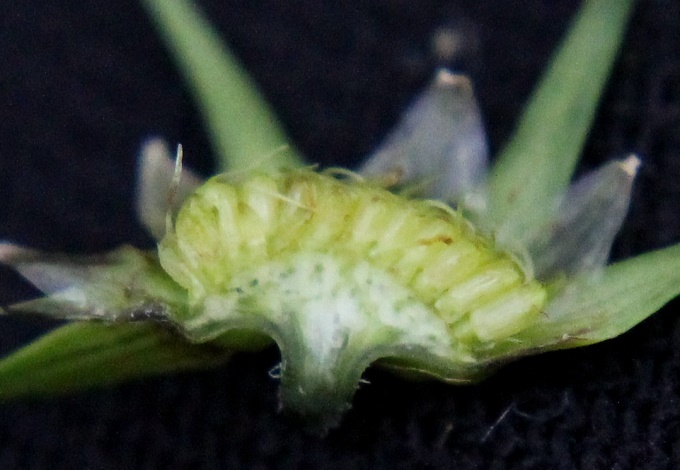


s-IB

l-IB

l-IB

IM

s-IB

l-IB

l-IB

F

E

D

RF

DF

l-IB

l-IB

l-IB

s-IB

s-IB

Fig S1 Different developmental states of wild-type and *gh* inflorescence

A-C, Different developmental states of wild-type inflorescence; D-F, Different developmental states of *gh* inflorescence; l-IB, leathery involucral bract; s-IB, semilucent membranous involucral bract; DF, disc flower; RF, ray flower. Scale bars = 2 mm in A, B, D; scale bars = 500 μm in C, E, F.

s-IB

s-IB

RF

l-IB

RF

DF

l-IB

DF

C

B

A
