# Supplementary material for: Reduced Expression of CbUFO Is Associated with the Phenotype of a Flower-Defective Cosmos bipinnatus
Source: Int J Mol Sci. 2019 May 21;20(10):2503. doi: 10.3390/ijms20102503 (PMC6566773; doi:10.3390/ijms20102503)
Supplement: Supplementary file 1 [file ijms-20-02503-s001.zip › supplementary files/Fig S2 Quantitative analysis of Involucral bracts.docx]

A


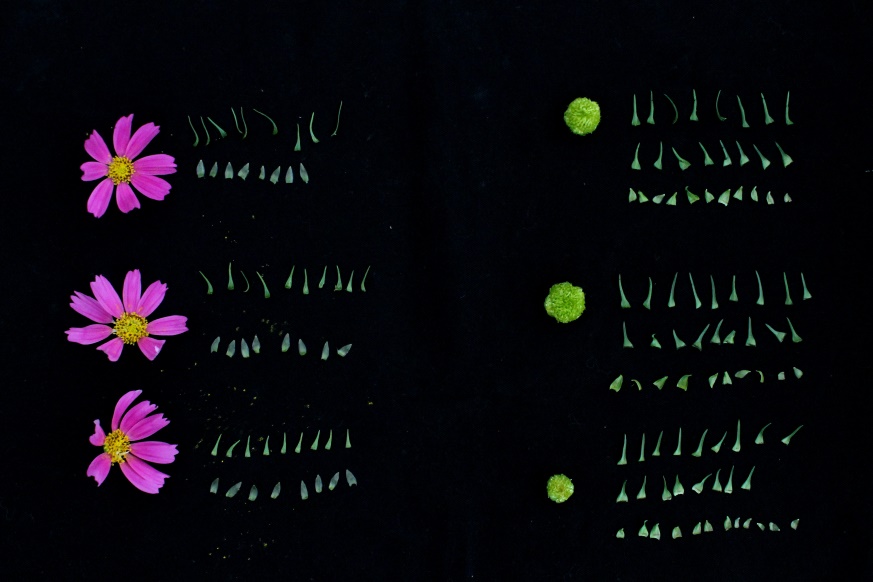


B

Fig S2 Quantitative analysis of Involucral bracts in wild-type and *gh* cosmos

A. Leathery involucral bracts in *gh* increased significantly compared to wild type

B. Examples of the number of bracts in some wild-type (left) and *gh* (right) cosmos

LB= Leathery involucral bracts; MB= membranous involucral bracts; scale bars = 1cm
