# Supplementary material for: Reduced Expression of CbUFO Is Associated with the Phenotype of a Flower-Defective Cosmos bipinnatus
Source: Int J Mol Sci. 2019 May 21;20(10):2503. doi: 10.3390/ijms20102503 (PMC6566773; doi:10.3390/ijms20102503)
Supplement: Supplementary file 1 [file ijms-20-02503-s001.zip › supplementary files/FIG S3Stereomicroscope observation.docx]

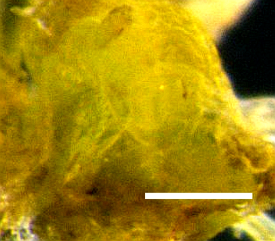

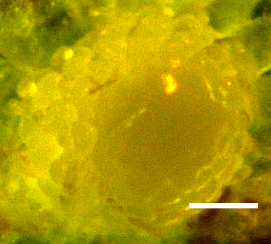

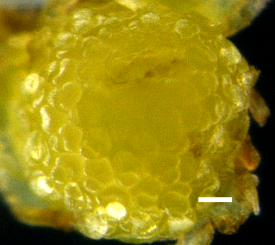

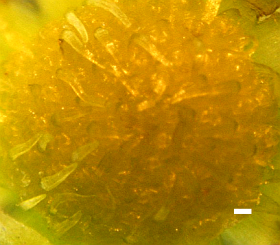


D

C

B

A


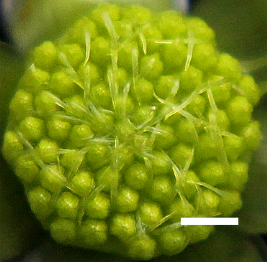

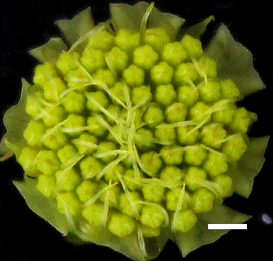

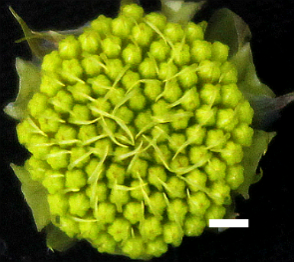

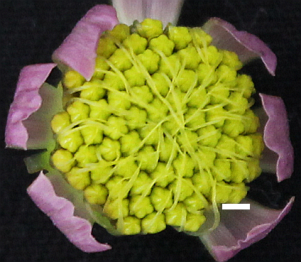


E

F

G

H


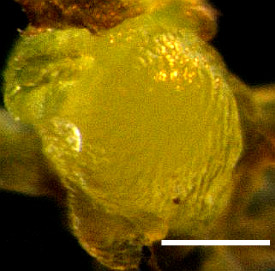

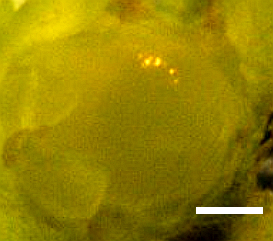

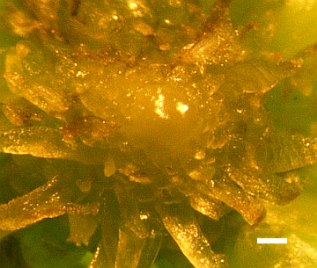

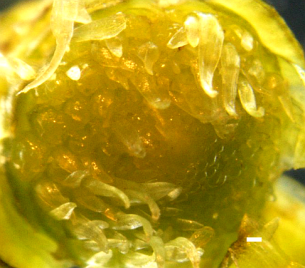


I

J

K

L


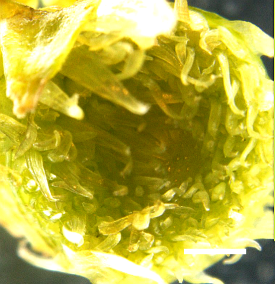

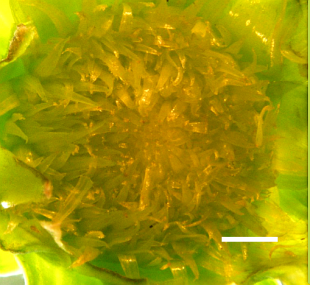

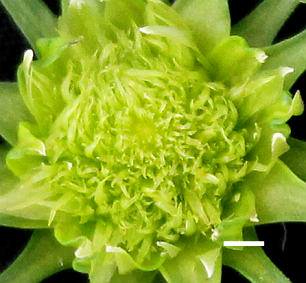

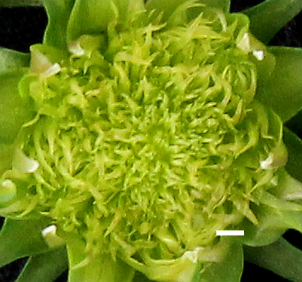


M

N

O

P

Fig. S3 Stereomicroscope observation of wide-type and *gh* cosmos.

A-H, Different stages of capitula in wide-type cosmos; I-P, Different stages of capitula in *gh* cosmos. Scale bars = 200 μm in A-D and I-L; Scale bars =1 mm in E-H and M-P
