# Supplementary material for: Reduced Expression of CbUFO Is Associated with the Phenotype of a Flower-Defective Cosmos bipinnatus
Source: Int J Mol Sci. 2019 May 21;20(10):2503. doi: 10.3390/ijms20102503 (PMC6566773; doi:10.3390/ijms20102503)
Supplement: Supplementary file 1 [file ijms-20-02503-s001.zip › supplementary files/FIG S4- Sequence alignments.docx]

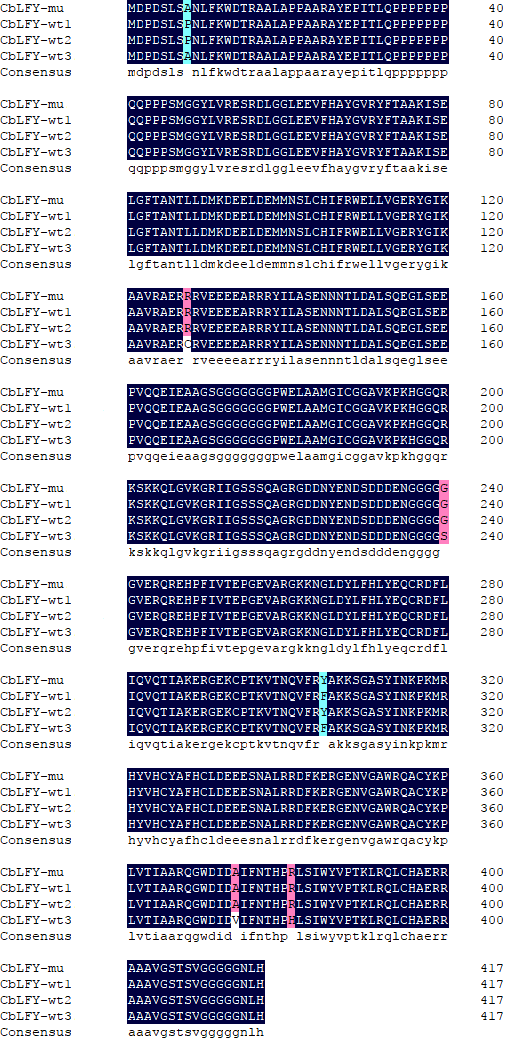


A


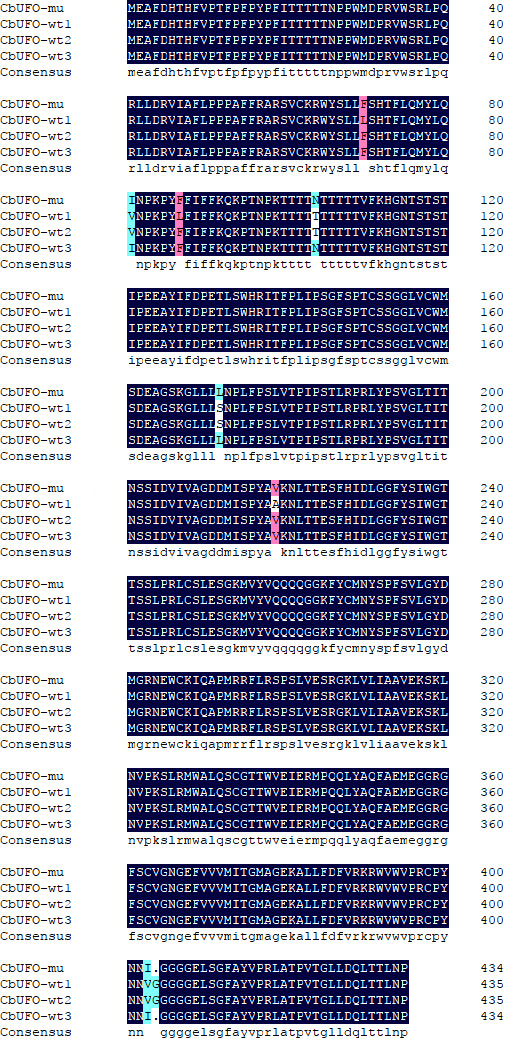


B

Fig. S4 Sequence alignments of *CbLFY* (A) and *CbUFO* (B) genes from different lines of wide-type and mutant cosmos.
