# Supplementary material for: Reduced Expression of CbUFO Is Associated with the Phenotype of a Flower-Defective Cosmos bipinnatus
Source: Int J Mol Sci. 2019 May 21;20(10):2503. doi: 10.3390/ijms20102503 (PMC6566773; doi:10.3390/ijms20102503)
Supplement: Supplementary file 1 [file ijms-20-02503-s001.zip › supplementary files/Fig S5.docx]

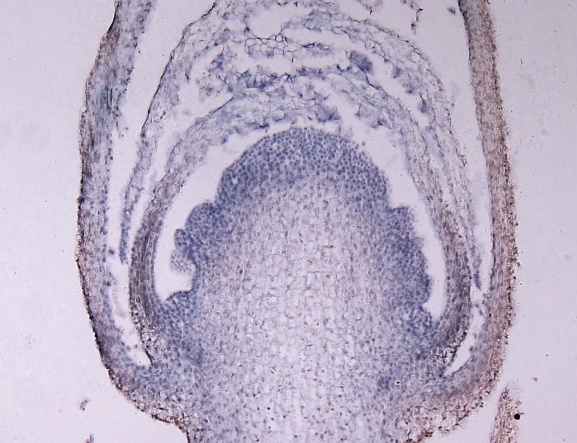

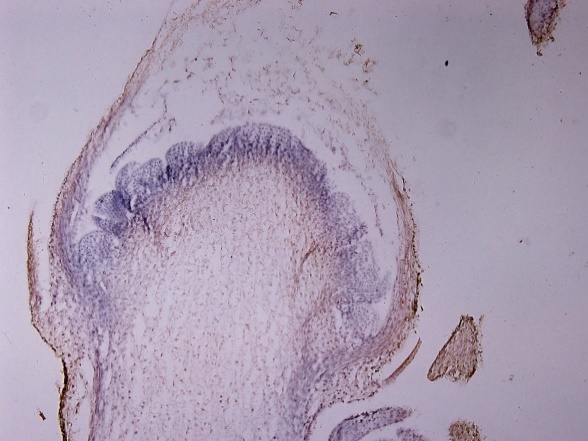

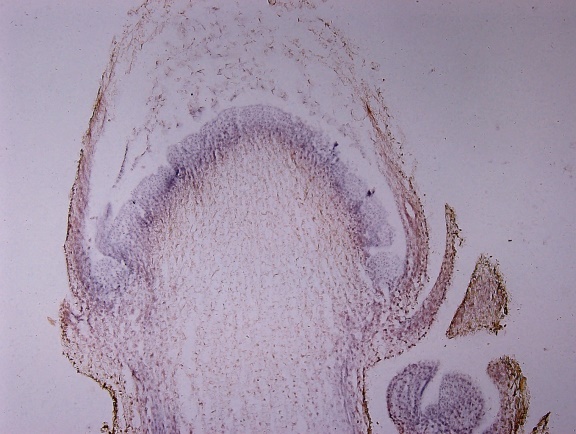

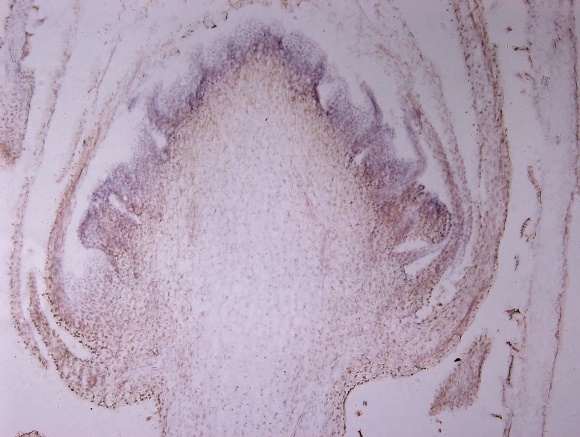


A

B

C

D

IB

IB

IB

IB

IB

RF

RF

RF

RF

DF

DF

DF

DF

IM

IM

IM

RF

Fig. S5 *In situ* hybridization of *CbUFO* in early development of wild-type inflorescence.

A, Early developmental stage of inflorescence; B-C, middle developmental stage of inflorescence; D, Late developmental stage of inflorescence. IM, inflorescence meristem; IB, involucral bract; DF, disc floral meristem; RF, ray floral meristem. Scale bars = 200 μm.
