# Supplementary material for: Reduced Expression of CbUFO Is Associated with the Phenotype of a Flower-Defective Cosmos bipinnatus
Source: Int J Mol Sci. 2019 May 21;20(10):2503. doi: 10.3390/ijms20102503 (PMC6566773; doi:10.3390/ijms20102503)
Supplement: Supplementary file 1 [file ijms-20-02503-s001.zip › supplementary files/table S1.docx]

**Table S1** Phenotypes of the transgenic *Arabidopsis* plants over-expressing *CbLFY* (A) and *CbUFO* (B), and the primer sequences used for the transgene expression analysis (C).

T_2_ transgenic lines whose progeny fitted a 3:1 segregation ratio of kanamycin resistance were chosen to record flowering time and floral phenotype. Values are mean ± SD (n=30). Symbols indicated significantly difference in comparison with Col-0 (*P<0.05 and **P<0.001)

| **Lines** | **Cauline leaves (numbers)** | **Rosette leaves (numbers)** | **Bolting time (days)** |
| --- | --- | --- | --- |
| CK | 2.3±0.5 | 12.5±0.6 | 27.1±1.1 |
| *CbLFY-5* | 2.1±0.5 | 7.8±1** | 20.2±1.1** |
| *CbLFY-7* | 2.1±0.5 | 8.8±0.7** | 22±1** |
| *CbLFY-12* | 2.2±0.5 | 8.8±0.7** | 21.8±1** |

**A**

| **Lines** | **Cauline leaves (numbers)** | **Rosette leaves (numbers)** | **Bolting time (days)** |
| --- | --- | --- | --- |
| CK | 2.3±0.5 | 12.5±0.6 | 27.1±1.1 |
| *CbUFO-6* | 2.2±0.5 | 12.8±0.7 | 27±0.9 |
| *CbUFO-8* | 2.2±0.6 | 12.8±0.7 | 26.8±0.9 |
| *CbUFO-16* | 2.2±0.5 | 13.1±0.8 | 27.2±0.9 |

**B**

**C**

| **Primer** | **sequence** |
| --- | --- |
| AtEF1a-F1 | GATTGACAGGCGTTCTGGTAAG |
| AtEF1a-R1 | CAGTCTGCCTCATGTCCCTCAC |
| CbLFY-DLF03 | GGATGCGTTATCTCAAGAGGGT |
| CbLFY-DLR03 | CTGTTTCTTGCTCTTCCGTTGG |
| CBUFO-DL03F | TGCAGTGGAGAAGAGTAAGCTG |
| CBUFO-DL03R | CCACAAACTCACCATTTCCGAC |
