# Supplementary material for: Reduced Expression of CbUFO Is Associated with the Phenotype of a Flower-Defective Cosmos bipinnatus
Source: Int J Mol Sci. 2019 May 21;20(10):2503. doi: 10.3390/ijms20102503 (PMC6566773; doi:10.3390/ijms20102503)
Supplement: Supplementary file 1 [file ijms-20-02503-s001.zip › supplementary files/table S2.docx]

Table S2 Primers using in the experiments

A, Primer sequences for isolation of *CbLFY* and *CbUFO*. B, Primer sequences for real-time RT-PCR analysis. C, Primer sequences for the preparation of RNA probes using *in situ* hybridization analysis.

| **Primer** | **sequence** |
| --- | --- |
| CBLFY-f1 | GAGCCWGTGCAACAAGARAWTG |
| CBLFY-r1 | GAACATAGTGTCTCATTTTCGG |
| cblfy-race-f1 | GGTATATTTTGGCTTCGGAGAA |
| cblfy-race-f2 | TGAAGCTGCGGGTAGTGGTGGT |
| RACE-3'long | ATTCTAGAGGCCGAGGCGGCCGACATG(T)30VN |
| RACE-3'shot | ATTCTAGAGGCCGAGGCGGCCGACATG |
| CbLFY-t1 | GCATATCCCCATTGCTGCTAGCTCCC |
| CbLFY-t2 | CCCACCTCCACCTCCACCACCACTAC |
| CbLFY-t3 | GCTTCAATCTCTTGTTGCACAGGCTC |
| CbLFYqc01F | AAGCCCCAAACAAACACAAGTC |
| CbLFYqc01R | TTTCCACATCCCACAAACTAAAC |
| CBUFO-f1 | AYTGKTTCHTNTTCTTYAARCA |
| CBUFO-r1 | GGVTCATAMSCAAANCCVTGCA |
| 1286-ufo-race-1 | TTTTATTCCATCTGGGGTACTA |
| 1286-ufo-race-2 | GGGCAAATTCTACTGCATGAACT |
| CbUFO-tail-1 | ACCACTCGTTTCGTCCCATATCAT |
| CbUFO-tail-2 | TTTGCCCCCTTGTTGTTGTTGGAC |
| CbUFO-tail-3 | TCAATGTGAAAGCTTTCAGTGGTT |
| CbUFOqc01F | CTCCCACTTCCACTTCCACTAC |
| CbUFOqc01R | GCTACTACAAGTGCAACCTAGC |

A

B

| **Primer** | **sequence** |
| --- | --- |
| Cbef1a-DLF1 | ACGCTCTTCTTGCTTTCACTCT |
| Cbef1a-DLR1 | TTGGCACGAATGGGATTTTGTC |
| CbLFY-DLF03 | GGATGCGTTATCTCAAGAGGGT |
| CbLFY-DLR03 | CTGTTTCTTGCTCTTCCGTTGG |
| CBUFO-DL03F | TGCAGTGGAGAAGAGTAAGCTG |
| CBUFO-DL03R | CCACAAACTCACCATTTCCGAC |

C

| **Primer** | **sequence** |
| --- | --- |
| CbLFY-tz300-F | ATATTTTCCGGTGGGAGTTGCT |
| CbLFY-tz300-R | CCAAGCTGTTTCTTGCTCTTCC |
| CbLFY-tz300-t7-F | TGTAATACGACTCACTATAGGGATATTTTCCGGTGGGAGTTGCT |
| CbLFY-tz300-t7-R | TGTAATACGACTCACTATAGGGCCAAGCTGTTTCTTGCTCTTCC |
| CbUFO-TZ-F | GTACGTCCAACAACAACAAGGG |
| CbUFO-TZR | GCTAAAACGGATTGAGGGTAGTCA |
| CbUFO-TZ-FT7 | TGTAATACGACTCACTATAGGGGTACGTCCAACAACAACAAGGG |
| CbUFO-TZRT7 | TGTAATACGACTCACTATAGGGCTAAAACGGATTGAGGGTAGTCA |
