# Supplementary material for: Reduced Expression of CbUFO Is Associated with the Phenotype of a Flower-Defective Cosmos bipinnatus
Source: Int J Mol Sci. 2019 May 21;20(10):2503. doi: 10.3390/ijms20102503 (PMC6566773; doi:10.3390/ijms20102503)
Supplement: Supplementary file 1 [file ijms-20-02503-s001.zip › supplementary files/Table S3.docx]

Table S3 Nucleic acid sequences and amino acid sequences in this study

Nucleic acid sequence of *CbLFY*:

ATGGACCCTGACTCACTCTCACCCAACTTGTTCAAGTGGGACACACGAGCCGCTTTAGCTCCACCGGCGGCTCGTGCGTACGAGCCGATAACCCTACAACCACCACCACCACCCCCACCGCAACAACCACCACCGTCCATGGGTGGCTATTTGGTGCGAGAAAGCCGGGATCTTGGTGGGCTTGAAGAGGTGTTTCATGCTTACGGCGTGAGGTACTTCACGGCGGCTAAGATATCAGAGCTCGGCTTCACGGCTAACACGCTGTTGGACATGAAAGATGAGGAGCTTGATGAGATGATGAACAGTTTGTGTCATATTTTCCGGTGGGAGTTGCTGGTGGGTGAGAGGTATGGGATTAAAGCGGCGGTGAGAGCCGAGCGACGCCGGGTGGAGGAGGAGGAGGCACGGCGGCGGTATATTTTGGCTTCGGAGAATAATAATACTCTGGATGCGTTATCTCAAGAGGGTTTGTCTGAAGAGCCAGTGCAACAAGAAATTGAAGCTGCGGGTAGTGGTGGTGGAGGTGGAGGTGGGCCATGGGAGCTAGCAGCAATGGGGATATGCGGAGGAGCCGTGAAACCGAAGCACGGTGGCCAACGGAAGAGCAAGAAACAGCTTGGTGTTAAGGGGAGAATAATTGGGTCATCTTCACAAGCGGGTAGAGGTGATGATAATTATGAGAATGATAGTGATGATGATGAGAATGGTGGTGGAGGCGGTGGTGTTGAGAGGCAACGCGAGCATCCGTTCATCGTGACGGAGCCGGGCGAGGTGGCACGAGGCAAAAAGAATGGACTTGATTACTTGTTTCATCTATATGAACAATGCCGTGATTTCTTGATTCAAGTTCAAACTATTGCTAAAGAGAGAGGTGAAAAATGTCCCACTAAGGTGACAAACCAGGTGTTTAGGTTTGCAAAGAAGTCTGGAGCTAGTTACATCAACAAGCCGAAAATGAGACACTATGTTCATTGCTATGCGTTTCATTGTCTCGATGAGGAGGAGTCGAATGCCCTAAGGCGGGATTTTAAGGAGAGAGGGGAGAATGTGGGGGCGTGGAGGCAGGCGTGCTACAAGCCGTTGGTGACGATTGCAGCACGACAAGGGTGGGACATTGATGCGATATTCAACACGCATCCACGCTTGTCGATATGGTATGTCCCGACTAAACTCCGCCAACTCTGTCATGCTGAACGTAGGGCGGCGGCTGTTGGATCGACGTCGGTTGGTGGTGGTGGTGGTAATCTGCATTTTTAG

Amino acid sequence of *CbLFY*:

MDPDSLSPNLFKWDTRAALAPPAARAYEPITLQPPPPPPPQQPPPSMGGYLVRESRDLGGLEEVFHAYGVRYFTAAKISELGFTANTLLDMKDEELDEMMNSLCHIFRWELLVGERYGIKAAVRAERRRVEEEEARRRYILASENNNTLDALSQEGLSEEPVQQEIEAAGSGGGGGGGPWELAAMGICGGAVKPKHGGQRKSKKQLGVKGRIIGSSSQAGRGDDNYENDSDDDENGGGGGGVERQREHPFIVTEPGEVARGKKNGLDYLFHLYEQCRDFLIQVQTIAKERGEKCPTKVTNQVFRFAKKSGASYINKPKMRHYVHCYAFHCLDEEESNALRRDFKERGENVGAWRQACYKPLVTIAARQGWDIDAIFNTHPRLSIWYVPTKLRQLCHAERRAAAVGSTSVGGGGGNLHF

Nucleic acid sequence of *CbUFO*:

ATGGAAGCTTTTGATCATACCCATTTTGTCCCCACCTTCCCCTTCCCCTATCCTTTCATCACCACCACCACCACCAATCCACCATGGATGGACCCCAGGGTCTGGAGCCGGCTTCCCCAACGCCTTCTCGACCGGGTCATCGCCTTCCTACCCCCTCCCGCCTTCTTCCGGGCCCGGTCCGTATGCAAAAGATGGTATTCCCTCTTGTTCTCTCACACTTTCCTCCAAATGTACCTCCAAGTCAACCCTAAACCCTACTTTTTCATCTTCTTTAAACAAAAACCCACAAACCCTAAAACAACAACCACCACCACCACCACTACCACTGTCTTCAAGCACGGTAACACTTCCACGTCCACCATCCCGGAGGAAGCTTACATCTTTGACCCCGAAACCCTCTCATGGCACCGTATCACCTTTCCCTTAATCCCATCAGGGTTTTCACCAACCTGCTCTTCAGGTGGACTCGTCTGCTGGATGTCAGACGAAGCGGGTTCGAAAGGACTGCTTCTTTCGAACCCGCTCTTTCCATCTTTAGTCACCCCTATACCTTCCACCTTAAGGCCTAGGTTATACCCTTCTGTAGGGTTAACCATCACAAACTCTTCAATCGATGTTATAGTAGCTGGAGATGACATGATATCTCCCTACGCCGTCAAGAACTTAACCACTGAAAGCTTTCACATTGACCTAGGTGGATTTTATTCCATCTGGGGTACTACTTCTTCTCTCCCAAGGCTATGCAGTTTAGAGTCTGGTAAAATGGTGTACGTCCAACAACAACAAGGGGGCAAATTCTACTGCATGAACTATAGTCCTTTTAGTGTGTTGGGTTATGATATGGGACGAAACGAGTGGTGCAAGATTCAAGCTCCGATGAGGAGGTTTTTAAGGTCTCCAAGCTTAGTGGAGAGCAGAGGGAAGCTGGTGTTGATAGCTGCAGTGGAGAAGAGTAAGCTGAATGTTCCTAAAAGTTTAAGGATGTGGGCACTGCAGAGCTGTGGGACAACATGGGTGGAGATTGAGAGGATGCCGCAGCAGTTGTATGCTCAGTTTGCAGAGATGGAAGGTGGGAGGGGGTTTAGTTGTGTCGGAAATGGTGAGTTTGTGGTGGTGATGATAACCGGGATGGCCGGTGAGAAGGCTTTGTTGTTTGATTTTGTTAGGAAGAGGTGGGTTTGGGTTCCTAGGTGTCCTTATAATAATGTTGGTGGTGGTGGTGGTGAGTTGAGTGGCTTTGCATATGTGCCTAGGCTTGCTACTCCGGTGACCGGACTTCTTGATCAGCTGACTACCCTCAATCCGTTTTAG

Amino acid sequence of *CbUFO*:

MEAFDHTHFVPTFPFPYPFITTTTTNPPWMDPRVWSRLPQRLLDRVIAFLPPPAFFRARSVCKRWYSLLFSHTFLQMYLQINPKPYFFIFFKQKPTNPKTTTTNTTTTTVFKHGNTSTSTIPEEAYIFDPETLSWHRITFPLIPSGFSPTCSSGGLVCWMSDEAGSKGLLLLNPLFPSLVTPIPSTLRPRLYPSVGLTITNSSIDVIVAGDDMISPYAVKNLTTESFHIDLGGFYSIWGTTSSLPRLCSLESGKMVYVQQQQGGKFYCMNYSPFSVLGYDMGRNEWCKIQAPMRRFLRSPSLVESRGKLVLIAAVEKSKLNVPKSLRMWALQSCGTTWVEIERMPQQLYAQFAEMEGGRGFSCVGNGEFVVVMITGMAGEKALLFDFVRKRWVWVPRCPYNNIGGGGELSGFAYVPRLATPVTGLLDQLTTLNPF

Partial nucleic acid sequence of *CbEF1α*:

CCCTTGTACCAGTCAAGGTTGGTTGACCTCTCAATCATGTTGTCACCCTCGAAACCAGAAATTGGCACGAATGGGATTTTGTCGGGGTTGTATCCGACCTTCTTCAAGTAAGAAGAAACTTCCTTCACGATTTCATCGTACCTAGCCTTCGAGTACTTGGGTGTGGTAGCATCCATCTTGTTACAACAGCAAATCATTTGCTTGACACCAAGAGTGAAAGCAAGAAGAGCGTGCTCACGGGTCTGACCGTCCTTAGAAATACCAGCCTCGAAACCACCAGTGGTGGAGTCAATAATCAAGACAGCACAATCAGCTTGTGAGGTACCAGTAATCATGTTCTTAATGAAATCACGATGTCCGGGGGCATCGATGACAGTGCAGTAGTACTTGGTGGTCTCAAACTTCCACAACGCAATATCGATGGTGATACCTCTTTCACGCTCTGCCTTAAGCTTGTCGAGCACCCATGCGTATTTGAAAGACCTCTTGTTCATCTCAGCTGCTTCCTTCTCAAAACGCTCAATGACACGCTTGTCGATACCTCCTAGCTTGTAGATCAAGTGA
