# Supplementary figures and images for: Reduced Expression of CbUFO Is Associated with the Phenotype of a Flower-Defective Cosmos bipinnatus
Source: Int J Mol Sci. 2019 May 21;20(10):2503. doi: 10.3390/ijms20102503 (PMC6566773; doi:10.3390/ijms20102503)

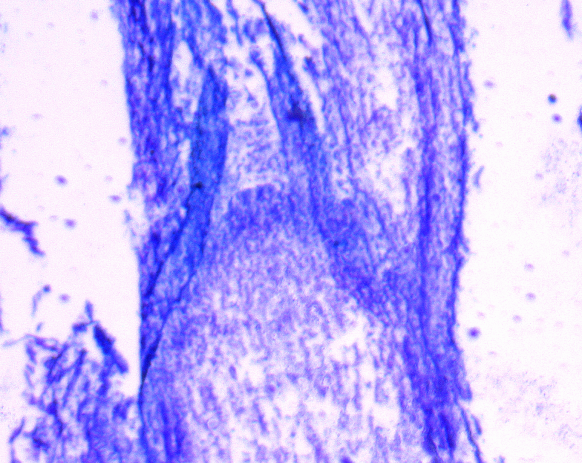

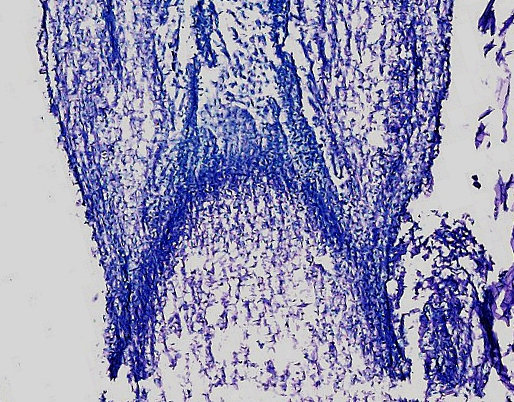


Fig. S6 Paraffin section of vegetative buds in wild type (left) and *gh* mutant (right).

Supplement: Supplementary file 1 [file ijms-20-02503-s001.zip › supplementary files/Fig S6 Paraffin section of vegetative buds in wild type.docx]
